# Complex within a Complex: Integrative Taxonomy Reveals Hidden Diversity in *Cicadetta brevipennis* (Hemiptera: Cicadidae) and Unexpected Relationships with a Song Divergent Relative

**DOI:** 10.1371/journal.pone.0165562

**Published:** 2016-11-16

**Authors:** Thomas Hertach, Stéphane Puissant, Matija Gogala, Tomi Trilar, Reto Hagmann, Hannes Baur, Gernot Kunz, Elizabeth J. Wade, Simon P. Loader, Chris Simon, Peter Nagel

**Affiliations:** 1 Department of Environmental Sciences, Biogeography, University of Basel, Basel, Switzerland; 2 Naturhistorisches Museum der Burgergemeinde Bern, Department of Invertebrates, Bern, Switzerland; 3 Muséum–Jardin des Sciences, Mairie de Dijon, Dijon, France; 4 Institut de Systématique, Évolution, Biodiversité, Muséum national d'Histoire naturelle, Sorbonne Universités, Paris, France; 5 Slovenian Academy of Sciences and Arts, Ljubljana, Slovenia; 6 Slovenian Museum of Natural History, Ljubljana, Slovenia; 7 University of Bern, Institute of Ecology and Evolution, Bern, Switzerland; 8 Department of Zoology, Karl Franzens University of Graz, Graz, Austria; 9 Department of Ecology and Evolutionary Biology, University of Connecticut, Storrs, Connecticut, United States of America; 10 United States Department of Agriculture, Center for Medical, Agricultural and Veterinary Entomology, Gainesville, Florida, United States of America; 11 Life Sciences Department, University of Roehampton, London, United Kingdom; Leibniz-Institute of Freshwater Ecology and Inland Fisheries, GERMANY

## Abstract

Multiple sources of data in combination are essential for species delimitation and classification of difficult taxonomic groups. Here we investigate a cicada taxon with unusual cryptic diversity and we attempt to resolve seemingly contradictory data sets. Cicada songs act as species-specific premating barriers and have been used extensively to reveal hidden taxonomic diversity in morphologically similar species. The Palaearctic *Cicadetta montana* species complex is an excellent example where distinct song patterns have disclosed multiple recently described species. Indeed, two taxa turned out to be especially diverse in that they form a “complex within the complex”: the *Cicadetta cerdaniensis* song group (four species studied previously) and *Cicadetta brevipennis* (examined in details here). Based on acoustic, morphological, molecular, ecological and spatial data sampled throughout their broad European distribution, we find that *Cicadetta brevipennis* s. l. comprises five lineages. The most distinct lineage is identified as *Cicadetta petryi* Schumacher, 1924, which we re-assign to the species level. *Cicadetta brevipennis litoralis* Puissant & Hertach ssp. n. and *Cicadetta brevipennis hippolaidica* Hertach ssp. n. are new to science. The latter hybridizes with *Cicadetta brevipennis brevipennis* Fieber, 1876 at a zone inferred from intermediate song patterns. The fifth lineage requires additional investigation. The *C*. *cerdaniensis* and the *C*. *brevipennis* song groups exhibit characteristic, clearly distinct basic song patterns that act as reproductive barriers. However, they remain completely intermixed in the Bayesian and maximum likelihood COI and COII mitochondrial DNA phylogenies. The closest relative of each of the four *cerdaniensis* group species is a *brevipennis* group taxon. In our favoured scenario the phylogenetic pairs originated in common Pleistocene glacial refuges where the taxa speciated and experienced sporadic inter-group hybridization leading to extensive introgression and mitochondrial capture.

## Introduction

Species delimitation and classification are among the most enduring contributions to science, and knowledge of species and subspecies boundaries is essential for conservation and management decisions. However, taxonomists are still far from a consensus on the definition of “species” [1], and many species concepts are conflicting or impractical to apply. In this study we investigate a European cicada complex (Insecta: Hemiptera: Cicadidae) that illustrates many problems raised in the species concept/species delimitation debates. From previous studies, we know that described species in this complex are morphologically almost indistinguishable and molecularly interdigitated. Behaviour, represented by acoustic song patterns, is similar in cases with distinct mitochondrial haplotypes; but song patterns are clearly different in some taxa that possess closely related or identical haplotypes [2,3]. In this complicated system, we attempt to revise the taxonomy in light of modern species concepts and address the question of how such a strange phylogenetic pattern might have evolved.

### Species concepts and integrative taxonomy

De Queiroz [4] recognized the primary defining criterion of species as “separately evolving metapopulation lineages” and suggested that evidence for speciation could come from one or more sources (e.g. reproductive isolation, diagnosability, monophyly). Increasingly, new studies indicate that speciation can occur in the presence of intermittent or continuous gene flow between diverging populations (e.g. [5–8]) and that species contain genes assembled from multiple semi-independently evolving lineages [9]. A useful new way of thinking about speciation was suggested by Butlin *et al*. [10]. In this view, reproductive isolation takes place in three phases: initiation, strengthening and completion. During each of these phases diverging populations can experience gene flow on a continuum from zero to high and can intermittently be allopatric, parapatric, or sympatric in no particular order. Thus gene flow can start and stop at various stages of the speciation process resulting in complex genetic and geographic patterns.

As noted by De Queiroz [4], species delimitation and conceptualization are two different issues. The variety of methods used to delimit species has rapidly increased with molecular based methods and models [11–13]. Recently, species boundaries have been estimated on genetic data exclusively (e.g. [14,15]). Raising concern over purely genetic definitions of species, a number of studies have demonstrated that gene trees do not reflect species boundaries (e.g. [16–19]) due to incomplete lineage sorting and ongoing gene flow (as reviewed in [20]).

Integrative taxonomy combines data from multiple sources such as genetics, morphology and ecology and has been increasingly applied to difficult groups [21,22]. It goes beyond naming species and provides insight into the speciation processes [21,23] and the recognition of current and past gene flow [12,13]. Results from various data sources conflict more often than generally expected. Only 41% of arthropod taxa from a literature survey showed broad agreement in delimitation among multiple data sets [21], thus interpretations seek to understand the source of the conflict. Here we use an integrative approach and combine acoustic, molecular, morphological, spatial and ecological data to understand the evolution of the *Cicadetta brevipennis* group.

### Bioacoustic relevance in cicadas

Cicada males produce well known songs with their timbals. These songs are species-specific, act as premating barriers to non-conspecific females, and are an important component of specific-mate recognition systems (SMRS; [24–26]). Acoustic characters have been used extensively to reveal hidden taxonomic diversity when morphological traits are missing or weak (e.g. [27–30]). Songs provide an excellent medium to study the early stages of reproductive isolation [31,32]. They are especially useful because even under scenarios of divergence with gene flow, genes involved in species recognition are less likely to cross species boundaries than other genes [33,34]; however although songs are the most probable indicators of species identity, a species-specific song does not guarantee that foreign genes are absent [32].

*Cicadetta montana* s. l. (Scopoli, 1772) is an ideal model system for bioacoustic research. As recently as the turn of the millennium [28,35,36], it has been successively shown to comprise a species complex of at least 13 morphologically similar but acoustically distinct species and two subspecies [2,37]. The faint high frequency calling songs possess a remarkable richness and complexity in rhythms.

### *Cicadetta brevipennis* and the *Cicadetta cerdaniensis* group

*Cicadetta brevipennis* Fieber, 1876 (sensu [28]) and *Cicadetta cerdaniensis* s. l. Puissant & Boulard, 2000 are widespread taxa belonging to the *Cicadetta montana* species complex. Recent research on the two taxa disclosed hidden diversity and confusing phylogenetic relationships, which we systematically attempted to comprehend with our investigations. Prior to the research presented here, *C*. *brevipennis* was viewed as one species (e.g. [38–41]) characterized by a distinct song pattern [28]: A binary structure of a long swelling echeme, followed after a brief break by a short echeme, is repeated multiple times.

In *Cicadetta cerdaniensis* s. l. some species diversity was more obvious and disclosed by a series of studies. In the first step, *Cicadetta cerdaniensis* s. l. was recognized as a group of different taxa characterized by the group-typical basic repetition of shorter echemes importantly modulated in power and species-specific, additional elements of various complexities [42,43]. Two species were described with qualitatively different songs: *Cicadetta cantilatrix* Sueur & Puissant, 2007 and *Cicadetta anapaistica* Hertach, 2011.

In a second step, the addition of molecular studies of the entire *Cicadetta montana* species complex [3] demonstrated a phylogenetically close relationship between the *Cicadetta cerdaniensis* song group and *Cicadetta brevipennis*. In particular, they comprised two interdigitated (polyphyletic), well supported main clades based on analysis of segments of the mitochondrial genes cytochrome C oxidase subunits I and II (COI and COII): “*cerdaniensis*-A + *brevipennis*-A” and “*cerdaniensis*-B + *brevipennis*-B + *C*. *anapaistica*” [3]. Some haplotypes were shared between song-divergent taxa and contamination was ruled out by re-sequencing. Nuclear markers (*elongation factor 1-alpha* and *period*) had a low number of variable and parsimony-informative sites and were informative only at the deeper levels of the tree. Thus molecular species delimitation methods (general mixed Yule-coalescent (GMYC; [15]) and Bayesian phylogenetics and phylogeography (BPP; [44])) were successful in recognizing some species (e.g. *Cicadetta montana* s. str.) but not the species in the *cerdaniensis-brevipennis* polytomy [3].

In a third step, in-depth investigations of “*cerdaniensis*-A” and “*cerdaniensis*-B” revealed a new species, *Cicadetta sibillae* Hertach & Trilar, 2015. In addition to having different mitochondrial haplotypes, it is distinguishable by quantitative acoustics, minor colouration characters, and geographic location [2].

The findings in these second and third steps of our series of studies stimulated the central question of this manuscript: Does *C*. *brevipennis* also comprises more than one taxon? Or more specifically: Are “*brevipennis*-A” and “*brevipennis*-B” two different species? Alternatively, one or both units could result from gene exchange with *cerdaniensis*-group taxa (e.g. introgression with mtDNA capture; [19,20,32]). “*Brevipennis*-B” could for example be introgressed with its neighbour *C*. *sibillae* (= former “*cerdaniensis*-B”). Our preliminary results, based on in-depth fieldwork suggested not only the two lineages “*brevipennis*-A” and “*brevipennis*-B” but rather a total of five potentially separately evolving metapopulations lineages [4], subsequently defined as operational taxonomic units (OTUs): brev, hipp, lito, petr and bulg. We treat bulg only tentatively.

We postulate that song differences between *C*. *brevipennis* s. l. and the *cerdaniensis* song group are such important and effective reproductive barriers that we can focus separately on the details of differentiation inside the *cerdaniensis* group [2] and now inside the supposed *C*. *brevipennis* group. All of the single-copy well developed, nuclear markers available for cicadas [45] are more appropriate for older divergences than are examined here. We therefore make use of other relevant nuclear-encoded data such as acoustics and morphology to evaluate the mitochondrial gene phylogeny and interpret the species histories. We study *C*. *brevipennis* s. l. from central Spain in the west to central Germany in the north, to the Black Sea in the east and to southern Italy in the south. We later combine the *C*. *brevipennis* and *cerdaniensis* group results and discuss the evolutionary histories based on a biogeographical scenario. We also discuss habitat preferences and potential threats to these taxa.

## Materials and Methods

### Fieldwork

Fieldwork for acoustic, molecular and morphological data collection was conducted in many European countries with a focus on Italy, France, Switzerland and Slovenia (S1 Table and S2 Table). Cicadas were localized with directional microphones. Several authors used ultrasonic detectors (e.g. Pettersson D 200) to enhance their ability to detect high frequency domains (10–20 kHz) over larger distances. Calling songs were recorded with different professional portable recorders (e.g. Marantz PMD 660) under natural conditions and over a wide temperature range (17 to 33°C). We measured the temperature of the surface, where the singing individual was presumed to sit, with a TFA ScanTemp 410 infrared thermometer [2] because duration of song elements can be strongly affected by temperature [46].

Voucher specimens were collected with a net. Cicadas are not protected by law in the investigated countries (with the exception of Spain); in biological reserves we obtained specific permits to collect specimens (S3 Table). Endangered taxa were collected conservatively, in some cases over several years (Torreilles, Monti della Daunia), so as not to endanger the survival of local populations. One leg was removed from selected fresh specimens and preserved in ethanol for later molecular analysis. All specimens were pinned and dried.

### Song analyses

**Fig 1:**
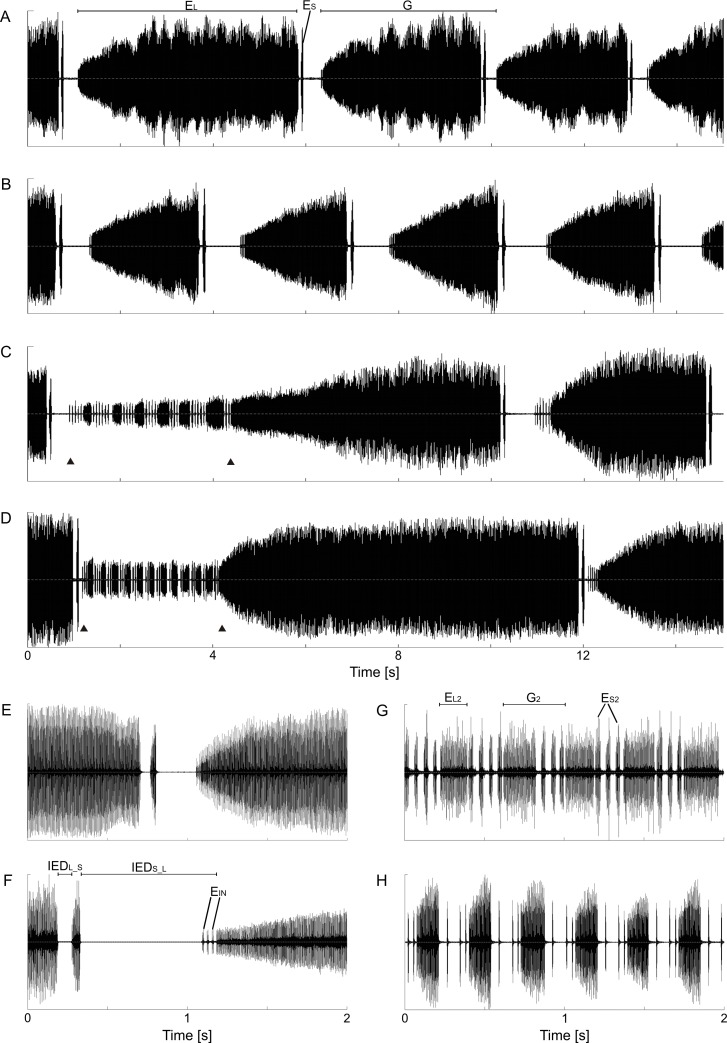
Oscillograms of typical calling songs of operational taxonomic units (petr, brev, hipp and lito) with terms of variables. (A)/(E) petr (Ticino, Switzerland/Kyffhäuser, Germany); (B)/(F) brev (central Slovenia); (C)/(G) hipp (Monti della Daunia, Italy); (D) lito (Torreilles, France). (H) *Tettigettula pygmea* (Olivier, 1790) song pattern for comparison to (G). (A) to (D) 15 s sections, (E) to (H) 2 s sections vs. amplitude. PH_1_ = phase 1 (A-F), PH_2_ = phrase 2 (C-D, start and end marked with triangles, and G); E_L_ and E_S_ = echeme types “long” and “short” of PH_1_; IED_L_S_ and IED_S_L_ = inter-echeme durations between echeme types E_L_ and E_S_ respectively E_S_ and E_L_; E_IN_ = introductory short echemes in front of the E_L_ (PH_1_); E_L2_ and E_S2_ = echeme types “long” and “short” of PH_2_; G and G_2_ = echeme/inter-echeme groups.

Songs were analysed using Raven Pro 1.4 (Cornell Lab of Ornithology). *Cicadetta brevipennis* s. l. calling songs consist of the repetition of a binary long-short-echeme pattern (e.g. [28]; Fig 1). This principle song element we call phrase 1 or main phrase (PH_1_). Puissant [47] described and illustrated a special courtship song with a fast progression of very short and longer echemes (under the name of *C*. *montana*). We call this part phrase 2 or “courtship element” (PH_2_). We use abbreviations for standard song variables (E = echeme, D = duration, F = carrier frequency and P = average power) which can be combined (e.g. ED = echeme duration).

**Fig 2:**
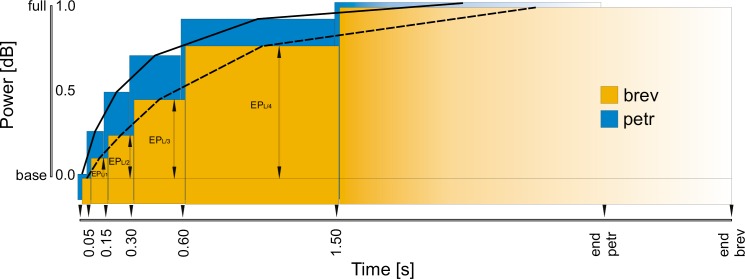
Schematic illustration of power levels (P) of the swelling long echemes (E_L_). Predefined time sections for average power measurement (base (EP_L/0_) ≔ 0–0.05 s, EP_L/1_ ≔ 0.05–0.15 s, EP_L/2_ ≔ 0.15–0.30 s, EP_L/3_ ≔ 0.30–0.60 s, EP_L/4_ ≔ 0.60–1.50 s, full power (EP_L/5_) ≔ 1.50 s to end). Values set to 0 for the baseline and 1 for the final time section. Example showing medium values of operational taxonomic units (OTU) brev and petr (n_ind_ = 35).

Acoustic recordings were assorted according to: 1) metapopulation membership; 2) quality of the recording; 3) the number of repeated elements (sample size); and 4) perch temperature. These categories were considered hierarchically for the choice of the next recording to be studied. Thus, we could avoid errors introduced by interesting traits in the song structure being included disproportionately by the analyser. Investigations were conducted on three sets of analyses: 1) The frequency of occurrence of the phrase 2 (163 males); 2) qualitative and quantitative aspects within phrase 1 (55 males); and 3) qualitative and quantitative aspects within the phrase 2 (31 males; all listed in S1 Table). For the first analysis, we counted the percentage of long-short echeme groups (PH_1_) followed by the phrase 2 (PH_2_). The minimal sample size was 10 long-short-echeme groups per individual. For the second analysis, we measured the time and carrier frequency domains of 20 long-short-echeme groups (G). The long echemes (E_L_) are characterized by a continuous increase of the power level. We investigated these progressions of power for the same 20 long echemes by splitting the echeme in predefined time steps (0–0.05 s, 0.05–0.15 s, 0.15–0.30 s, 0.30–0.60 s, 0.60–1.50 s, 1.50 s to end) and measuring the average power level for each segment (Fig 2; Raven function: “Average Power” in decibel, dB). The 0–0.05 s segment was defined as the baseline and the absolute dB values were set to the relative value 0 (or 0%). The absolute values of the 1.50 s to end segment were used as the maxima and correspondingly set to 1 (or 100%). We were then able to calculate relative power differences for each segment in between with respect to the baseline and maximum values. We obtained comparable results in the program Audacity calculating the Root Mean Square (RMS). We also investigated the number and duration of syllables (= complete cycles of timbal movements) forming the short echemes (see [2]). For the third analysis, the structure of the phrase 2 (PH_2_), we counted the number of short echemes (E_S2_) in between two neighbouring long echemes (E_L2_; Fig 1G), measured some duration characters and compared the power levels of E_L2_ echemes with the following E_L_ (1.50 s to end segment). The sample size was constant with six echeme/inter-echeme groups (G_2_; Fig 1G) (the forth, third and second last of the phrase) taken from two different PH_2_ per individual.

In a very few cases, we randomly concatenated song recordings from different males to create composite individuals in order to reach the minimal sample size of song elements. This is especially true for the Torreilles population in southern France. This practice may equalize extreme song patterns of single specimens towards the taxon-typical medium values but did not influence the final conclusion.

Statistical tests on song variables were conducted in R [48] between individuals of the two widespread operational taxonomic units (OTUs); i.e. brev and petr. Variables were tested for temperature dependencies between these two OTUs with Analysis of Covariance (ANCOVA; see [2]). ANCOVAs yielded ambiguous results for the temperature dependencies (several variables close to the significance level 0.05). However, they demonstrated that interactions between the covariate “temperature” and the categorical factor “taxon” are not present and rates of change (= slopes) could consequently be regarded as similar between the two OTUs. Therefore, we built up a general lineal model (GLM) to eliminate the supposed but uncertain temperature dependencies for each variable in the form: variable = a + b * temperature + c * OTU where the two OTUs were set to 0 and 1 (R commands: lm(variable~temperature+OTU) for normally distributed variables and glm(…) for Poisson-distributed variables). Values of the partial residuals in the variance of the opposite OTU were scored as failures. Measurements of the remaining OTUs (i.e. hipp and lito) were afterwards assigned for each variable and individual to these variances and evaluated as fitting perfectly to the first or the second OTU or being located in the overlap. A Principal Component Analysis (PCA) was conducted to visualize the power of a combination of a few song characters for separating taxa. We used a correlation matrix implemented in the function prcomp() with the option “scale = TRUE”. Graphs were generated with the package ggplot2 [49] and illustrations of oscillograms produced with Seewave [50].

The song recordings are preserved in the Slovenian Wildlife Sound Archive of the Slovenian Museum of Natural History Ljubljana. Song examples are presented at the web pages *Songs of European singing cicadas* http://www.cicadasong.eu. Raw data of acoustic and other analyses were deposited at Figshare: doi: 10.6084/m9.figshare.3168250.

### Molecular phylogenetic analyses

For reconstructing phylogenies, a molecular dataset was assembled of the mitochondrial gene segments from the COI and COII genes. The dataset improves upon previous work [2,3] by increasing the number of specimens and sampling new geographic regions. The eight newly sequenced specimens of *C*. *brevipennis* s. l. originate from Eastern and Northern parts of the distribution and include a type specimen collected in 1916 (*C*. *montana* f. *petryi* Schumacher, 1924; Natural History Museum of Berlin). The total number of specimens of *C*. *brevipennis* s. l. is now 33. *Cicadetta montana* s. str. was chosen as an appropriate out-group in all analyses [3]. For each cicada, genomic DNA was extracted from one leg using a Qiagen DNeasy Blood & Tissue Kit, following the manufacturers’ instructions, except that DNA digestion was conducted over 24 hours or more. Polymerase chain reaction (PCR) amplification was performed with a PCR Beads kit (GE Healthcare) and the following primers: C1-J-2195 and TL2-N-3014 (COI) with an annealing temperature of 56°C and TL2-J-3034 and TK-N-3786 with an annealing temperature of 53 or 50°C (COII; [51]). PCR products were sequenced using the forward and reverse primers by the Sanger DNA sequencing service of Microsynth AG, Balgach, Switzerland. The complementary sequences were assembled and edited with CodonCode Aligner 4. Sequences were aligned with MUSCLE [52] in Geneious Pro 6.1.8 (http://www.geneious.com) with default settings. We constructed two alignments, one including all *C*. *brevipennis* s. l. sequences and one including additionally the closely related *cerdaniensis* song group sequences (67 samples including the out-group). We adopted this strategy to demonstrate relationships within the *C*. *brevipennis* s. l. and relationships on a broader taxonomic scale. For each alignment codon positions for protein coding genes were determined using TranslatorX [53]. Sequences are deposited in GenBank: COI: KT901699—KT901780 and KU679422—KU679433, COII: KT901473—KT901554 and KU679434—KU679445.

As in Hertach *et al*. and Wade *et al*. [2,3] COI and COII gene trees were concatenated and PartitionFinder 1.0.1 [54] was used to determine the best fitting model under the Bayesian Information Criterion. The program suggested a total of three partitions (both genes shared the same model for each codon position in each alignment) which were used for Bayesian Inference and Maximum Likelihood (ML) analysis. Bayesian Inference was carried out with MrBayes 3.2.1 [55] employing parallel runs of four simultaneous Markov chains for 10 million generations, sampling every 1000 generations. Model parameters were independently optimized for each partition. The first million generations were discarded as burn-in, based on stationarity of the log-likelihood tree scores, and whether the effective sample size of all parameters were > 200, evaluated using Tracer 1.5 [56]. We conducted ML analysis with non-parametric bootstrapping in RAxML 8.2 [57] under the same partitioning schemes applied in Bayesian Inference. We ran 1000 bootstrap replicates in ML analysis. Geneious Pro 6.1.8 was used to calculate genetic distances between samples.

### Morphological analyses

Song-identified males or females from single-species local populations were included in the morphological analyses, in total 132 dry prepared specimens of *C*. *brevipennis* s. l. were studied. The terminology of Moulds [58] is used for the descriptions. Some principal distances (body length, body width, fore wing length, fore wing width) were directly measured with vernier callipers or photographed with a Leica DFC425 camera on a Leica M205 C stereomicroscope (fore wing) or a Keyence VHX 2000 digital photo-microscope (body). These measurements are part of an ongoing comprehensive morphometric study on a larger number of *Cicadetta montana* s. l. taxa. In our current work, they are used to document variability among specimens for the taxonomic descriptions. ImageJ 1.47 [59] provides a simple application to measure distances from the photographs.

### Distribution patterns

Maps based on specimen localities were generated with ArcGIS (map source: http://www.worldclim.org and http://www.diva-gis.org). The final distribution map integrates previously published records from the literature and unpublished observations from the databases of the first four authors (S2 Table). We define metapopulations as a set of conspecific local populations that presumably interact via individuals moving among populations [60].

### Nomenclatural acts

We infer taxonomic conclusions under De Queiroz’ [4] “Unified Species Concept”, which led us to combine data sets from different sources in a practical way. The taxa have been checked for concordance with the numerous old names existing and intermediately regarded as synonyms of *Cicadetta montana* s. l. based on original descriptions, type localities and, whenever available, type specimens. We also critically examined the name *Cicadetta brevipennis* Fieber, 1876 with the aid of published and unpublished original works preserved in the National Museum of Natural History Paris (MNHN; Soulier-Perkins, pers. comm.) and putative type specimens according to the International Code of Zoological Nomenclature [61].

The electronic edition of this article conforms to the requirements of the amended International Code of Zoological Nomenclature, and hence the names of undescribed taxa contained herein are available under that Code from the electronic edition of this article. This published work and the nomenclatural acts it contains have been registered in ZooBank, the online registration system for the ICZN. The ZooBank LSIDs (Life Science Identifiers) can be resolved and the associated information viewed through any standard web browser by appending the LSID to the prefix “http://zoobank.org/”. The LSID for this publication is: urn:lsid:zoobank.org:pub:F60DFD71-87D6-40B0-AE63-4DDB38E37F34. The electronic edition of this work was published in a journal with an ISSN, and has been archived and is available from the following digital repositories: PubMed Central, LOCKSS.

## Results

Data from 78 *Cicadetta brevipennis* s. l. populations from Italy, France, Switzerland, Slovenia, Germany, Austria, Bulgaria, Romania, Croatia, Serbia and Spain were analysed. The operational taxonomic units (OTUs) brev, hipp and lito are subunits of the former “*brevipennis*-B”; petr is identical with “*brevipennis*-A”; bulg is completely new and the data set is not informative enough for conclusions. The five OTUs are colour-coded in the illustrations: brev = orange, hipp = red, lito = pink, petr = blue and bulg = green.

### Molecular phylogenetic analyses

**Fig 3:**
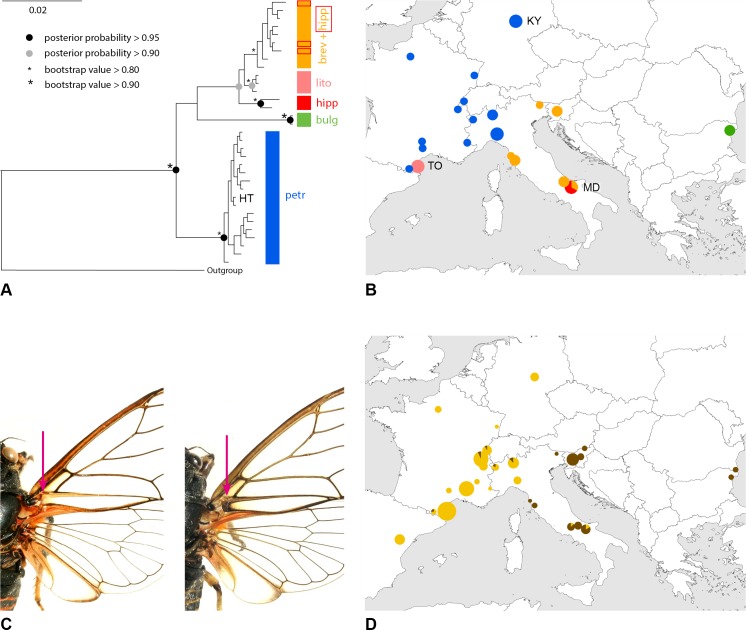
Molecular phylogenetic relationships and colouration trait within the *brevipennis* song group. (A) Bayesian mtDNA phylogeny with posterior probabilities (filled circles) and ML bootstrap values (star icons) from RAxML analysis; (B) geographic distribution of colour-coded clades in the phylogenetic tree; (C) dark or yellowish colour of basal junction of anal veins in the fore wing; and (D) geographic distribution of this colouration trait. Notes: Supposedly introgressed hipp specimens marked with red square. HT = holotype. Data is pooled for several closely neighbouring local populations in the maps. Size of the circles relative to the number of investigated specimens (n_max_ = 3 resp. 23). Important populations: KY = Kyffhäuser, MD = Monti della Daunia, TO = Torreilles.

The *Cicadetta brevipennis* song group forms three well supported main clades in the Bayesian concatenated COI and COII phylogeny (posterior probabilities 0.94–1; Fig 3A and 3B): petr, bulg and the remaining three OTUs. In Maximum Likelihood mitochondrial phylogeny petr and bulg have strong support, while the third branch is weaker (bootstrap values 0.76–1; Fig 3A and 3B and S2 Fig). This clade is again clustered in three subclades containing lito, hipp and brev/hipp (posterior probabilities 0.81–0.99, bootstrap values 0.81–0.89). It appears evident that the hipp unit was partly introgressed by brev since we found both distinct haplotypes in one single population (Monti della Daunia “MD”). The supposedly original hipp mtDNA is restricted to this location. The lito haplotypes are endemic to coastal southern France (Torreilles “TO”).

Average uncorrected pairwise genetic distances of the three main clades in the *brevipennis* song group are 2.1 to 3.0% (in COI) and 1.5 to 3.2% (in COII) (Table 1). From previous studies we know that genetic distances among any song delimited taxa in the *C*. *montana* complex are at most 5.1% for the two mitochondrial genes concatenated [3].

**Table 1:** Uncorrected average pairwise distances of mtDNA within the *Cicadetta brevipennis* song group (in %).

	brev	hipp 1	hipp 2	hipp (tot)	lito	petr	bulg
**brev**	***0*.*2/0*.*1***	1.3	0.4	0.8	0.7	2.6	2.6
**hipp 1**	0.6	***0*.*7/0*.*3***	1.5		0.7	3.0	2.8
**hipp 2**	0.1	0.7	***0*.*5/0*.*0***		0.8	2.7	3.0
**hipp (tot)**	0.3			***1*.*1/0*.*4***	0.7	2.9	2.9
**lito**	0.2	0.8	0.2	0.5	***0*.*0/0*.*0***	2.1	2.4
**petr**	1.6	1.9	1.6	1.7	1.5	***0*.*2/0*.*0***	2.4
**bulg**	2.8	3.2	3.0	3.0	3.1	3.0	***0*.*0/0*.*0***

COI above diagonal, COII below, within taxa distance bold and italic in the diagonal (COI/COII). OTU hipp has two clearly different haplotypes.

### Morphology

The majority of species within the *C*. *montana* species complex exhibit few interspecific morphological traits but remarkably high intraspecific variability. Lineages in the *brevipennis* group can be distinguished from each other with high probability by the predominantly yellowish versus dark basal junction of the anal veins in the fore wing; i.e. for petr (94%, n_ind_ = 72, males and females), lito (100%, n_ind_ = 23), brev (0%, n_ind_ = 19), hipp (11%, n_ind_ = 14) and bulg (0%, n_ind_ = 4; Fig 3C and 3D). Only ten of the 132 specimens examined morphologically were ambiguous: one was predominantly dark instead of yellowish; another yellowish instead of dark; and eight specimens were ambivalent dark and yellowish (counted half for each character state).

In hipp, the slightly elevated value of light junctions results from a distinct dimorphism: Some specimens in the south-easternmost Italian Monti Della Daunia (“MD”) population are generally paler. The two morphs in hipp are not correlated with the two haplotypes mentioned above. The specimens we designate as lito are generally short winged and with light wing venations and are distinguishable from many specimens within the *C*. *montana* species complex and in particular within the *brevipennis* group.

### Song patterns

All songs of the *Cicadetta brevipennis* group are composed of a repeated binary structure. A long echeme (E_L_) of approximately 3.5 s duration (but with extremes from 0.7 to 60 s) precedes a short pause (IED_L_S_) and a short echeme (E_S_) both of them lasting no longer than 0.1 s (Fig 1). In rare cases and more often in the courtship behaviour the E_S_ can be missing. Duration and carrier frequency measurements and counts are summarized in Table 2.

**Table 2:** Measurements of acoustic variables in the *Cicadetta brevipennis* song group for the entire temperature range.

		petr	brev	hipp	lito
**Phrase 1 (PH**_**1**_**)**		**(n**_**ind**_ **= 19)**	**(n**_**ind**_ **= 16)**	**(n**_**ind**_ **= 12)**	**(n**_**ind**_ **= 4)**
Perch temperature [°C]		24.7 ± 3.1	26.2 ± 3.0	23.6 ± 2.6	24.0 ± 0.0
Durations [s]	ED_L_	3.058 ± 1.121	3.798 ± 1.200	2.936 ± 0.839	***8*.*890 ± 5*.*985***
	ED_S_	***0*.*043 ± 0*.*006***	0.056 ± 0.008	0.055 ± 0.007	0.056 ± 0.011
	IED_L_S_	0.064 ± 0.012	0.073 ± 0.012	0.071 ± 0.009	0.080 ± 0.007
	IED_S_L_	***0*.*488 ± 0*.*198***	1.100 ± 0.328	0.956 ± 0.308	***0*.*311 ± 0*.*076***
Counts	E_IN_ number	***1*.*2 ± 0*.*7***	6.7 ± 5.0	7.4 ± 4.0	3.7 ± 0.5
	E_IN_ rate*	***10%***	76%	87%	88%
	Syllables/E_S_	6.1 ± 0.8	7.2 ± 1.1	7.6 ± 0.9	6.9 ± 0.4
E_L_ standardized power levels	EP_L/1_	***0*.*25 ± 0*.*04***	0.12 ± 0.04	0.14 ± 0.07	0.15 ± 0.04
	EP_L/2_	***0*.*48 ± 0*.*07***	0.25 ± 0.07	0.29 ± 0.11	0.33 ± 0.09
	EP_L/3_	***0*.*70 ± 0*.*07***	0.46 ± 0.08	0.52 ± 0.12	0.55 ± 0.12
	EP_L/4_	***0*.*91 ± 0*.*04***	0.77 ± 0.05	0.83 ± 0.09	0.78 ± 0.13
Frequency [kHz]	EF_L_ (centre)	14.3 ± 1.0	14.6 ± 0.8	14.4 ± 0.8	14.7 ± 0.2
	EF_L_ (1. Quartile)	13.4 ± 0.9	13.6 ± 0.7	13.4 ± 0.8	13.9 ± 0.2
	EF_L_ (3. Quartile)	15.5 ± 1.1	15.8 ± 0.9	15.2 ± 0.9	15.6 ± 0.1
	EF_S_	14.2 ± 1.0	14.5 ± 0.9	14.3 ± 0.8	14.7 ± 0.3
**Phrase 2 (PH**_**2**_**)**		**(n**_**ind**_ **= 4)**	**(n**_**ind**_ **= 4)**	**(n**_**ind**_ **= 17)**	**(n**_**ind**_ **= 6)**
Durations [s]	ED_2L_	0.220 ± 0.083	0.198 ± 0.071	0.205 ± 0.055	0.199 ± 0.052
	GD_2_	0.502 ± 0.066	0.446 ± 0.119	0.502 ± 0.107	0.433 ± 0.126
Counts	E_2S_	4.5 ± 0.6	4.5 ± 1.7	4.2 ± 0.9	***3*.*3 ± 1*.*1***
Power [dB]	EP_L/5_—EP_2L_	13.6 ± 1.6	16.4 ± 3.3	***10*.*6 ± 1*.*3***	14.4 ± 2.6

Mean values between individuals ± SD. n_ind_ = number of individuals. Putative hybrids excluded. Interesting values bold and italic. D = duration, F = frequency, P = power. For song variables see Figs 1 and 2.

* E_IN_ rate is the portion of long echemes fitting with the following minimal criterion: at least two introductory chirps in front of the long echeme followed by minimal pauses of 0.015 s.

**Fig 4:**
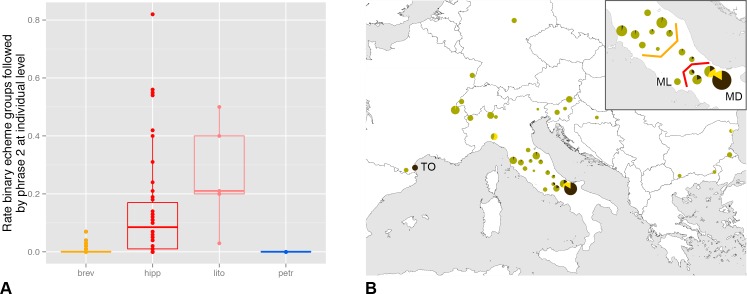
Phrase 2 (PH_2_) occurrences. Acoustic trait that separates hipp and lito from other OTUs. (A) PH_2_ used as calling song rather than courtship at the individual level and (B) as diagnostic trait on population level (dark brown = PH_2_ calling song, yellow = PH_2_ courtship song, olive = PH_2_ not emitted; values relative to the PH_2_ rate of the “MD” population). Orange and red lines mark suggested limits of OTUs brev and hipp, respectively, in Central Italy. Notes: Data is pooled for several closely neighbouring local populations. Size of the circles relative to the number of investigated individuals (n_max_ = 27). Populations mentioned in the text: MD = Monti della Daunia, ML = Monti Lepini, TO = Torreilles.

Song patterns show at least one obvious qualitative difference among taxa. In brev and petr populations, phrase 2 (PH_2_) is used for courtship as originally defined above. It is sung when a female is in the vicinity of the male. In lito and hipp populations, PH_2_ is emitted in a clearly elevated frequency of occurrence (Figs 1, 4A and 4B). We observed the highest rates in Torreilles (“TO”, lito), where 27% of the long-short echemes (n = 117) were followed by PH_2_, and at Monti Della Daunia (“MD”, hipp) with a 23% rate (n = 746). In these populations we hypothesize that PH_2_ forms part of the calling song to attract females. In addition to the frequency of occurrence, five other facts support this hypothesis: 1) Naturally and artificially completely isolated males sang PH_2_; 2) PH_2_ was emitted in the first calls in the morning when mating is unusual; 3) males singing PH_2_ often flew to new perches while males that are engaged with females are generally known to be more stationary; 4) we demonstrate that in hipp the PH_2_ is relatively louder in comparison with PH_1_ than in all other taxa (n_ind_ = 31), which is a good indication of longer distance communication (Wilcoxon rank sum test: W = 12, p < 0.001, Table 2 and S1 Fig); and 5) these differences hold up over time in the few cases where repeated measurements were made. Nevertheless, it was not possible in every case to infer whether PH_2_ was part of the courtship behaviour or of the calling song since not many recorded individuals were seen. This fact makes conclusions more difficult, especially in a contact zone between typical brev and typical hipp where the values are intermediate on the population level (Fig 4B, enlarged part).

**Fig 5:**
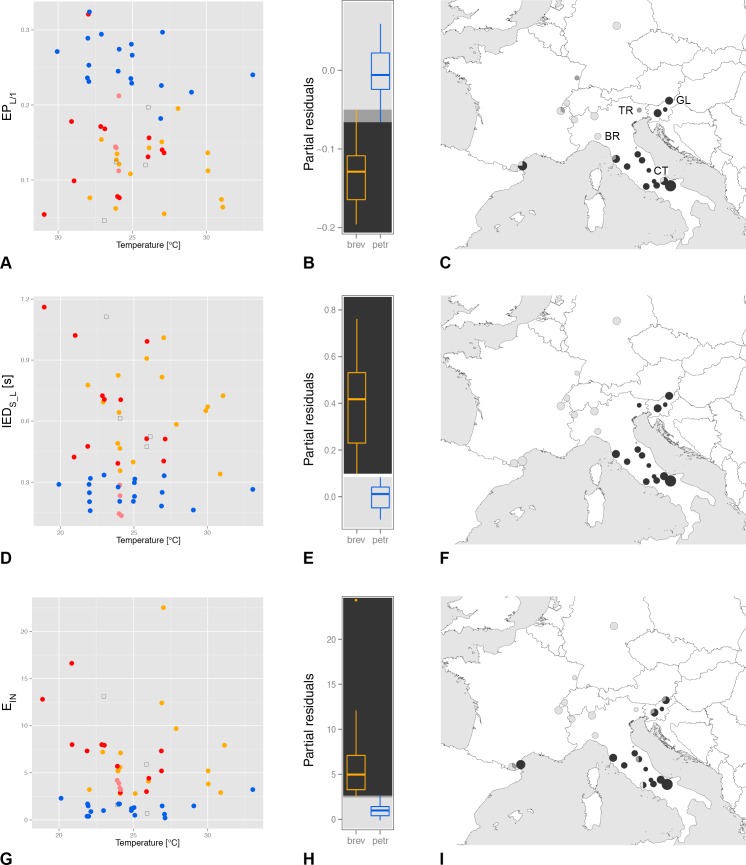
Three acoustic traits to distinguish petr from brev. (A-C) standardized power level EP_L/1_, (D-F) minimal duration of the IED_S_L_ and (G-I) number of introductory chirps (E_IN_). (A)(D)(G) dependencies on the perch temperature for single individuals (blue = petr, orange = brev, red = hipp, pink = lito, hollow squares = potential hybrids; temperature jitter = 0.15), (B)(E)(H) boxplots after controlling for the temperature with general linear models for brev and petr and (C)(F)(I) spatial patterns of temperature controlled values (light grey = typically petr, black = typically brev, dark grey = within the overlap). Notes: Data is pooled for several closely neighbouring local populations. Size of the circles relative to the number of investigated individuals (n_max_ = 6). Populations mentioned in the text: BR = Brallo di Pregola, CT = Campotosto, GL = Glanz an der Weinstrasse and TR = Travesio.

With in-depth observations and analyses, we became aware of additional song characters in the first and main phrases (PH_1_) that distinguish between the widespread OTUs brev and petr (Fig 5 and Table 2). All these characters have been tested for temperature dependency and significance first with ANCOVA and then with GLM (Table 3). 1) The progression of the power levels in the E_L_ is faster in petr populations than is typical for brev, resulting in a more parabolic than linear envelope of the amplitude (Figs 2 and 5A–5C). EP_L/1_ and EP_L/2_ are the most distinct. 2) IED_S_L_ (inter-echeme durations between the end of the E_S_ and the next E_L_) are shorter in petr than in brev. The minimum IED_S_L_ value per individual perfectly distinguishes brev from petr in our dataset (Figs 1E, 1F and 5D–5F). 3) OTU brev populations often start the E_L_ echemes with a series of stammering short chirps (E_IN_) while these chirps are much rarer in petr (Figs 1E, 1F and 5G–5I). In petr the chirps are somehow an artefact caused by starting problems of the song apparatus (e.g. low temperatures) while in brev they are likely to have become part of the specific-mate recognition systems. Almost as distinct as the number of chirps is the percentage of starts with at least two introductory echemes followed by pauses of at least 0.015 s (= minimum criterion, E_IN_ rate). 4) Sums of short pause and short echeme (IED_L_S_ + ED_S_) are significantly longer in brev than in petr. However, the overlap is higher than in the first three characters. 5) The speed of the timbal movements measured by the use of the syllable duration of E_S_ is significantly faster in petr than in brev. Summarizing, brev is quantitatively always more sluggish than petr.

**Table 3:** General linear models (GLM) testing for temperature dependencies and acoustic significances between petr and brev OTUs.

Variable	Model	t/z_temp_	p_temp_	t/z_OTUs_	p_OTUs_	a	b	c
**E**_**IN**_ **number**	glm	1.22	0.2230	6.88	0.0000	-0.662	0.036	1.622
**E**_**IN**_ **rate**	glm	-0.33	0.7450	2.54	0.0110	-1.490	-0.030	2.011
**EP**_**L/1**_	lm	-1.54	0.1350	-10.28	0.0000	0.335	-0.003	-0.134
**EP**_**L/2**_	lm	-2.12	0.0418	-9.85	0.0000	0.679	-0.008	-0.224
**IED**_**S_L**_ **(minimum)**	lm	-0.23	0.8230	7.75	0.0000	0.299	-0.002	0.396
**IED**_**L_S**_ **+ ED**_**S**_	lm	-2.87	0.0072	5.23	0.0000	0.162	-0.002	0.025
**Syllable duration**	lm	-4.21	0.0002	3.58	0.0011	0.012	-2.1x10^-4^	1.1x10^-3^

Function: variable = a + b * temperature + c * OTU where the two OTUs were set to 0 and 1. Significance and coefficients for 34 individuals. For song variables see Figs 1 and 2 and Table 2.

Individuals belonging to OTUs hipp and lito have first phrases 1 (PH_1_) that are similar to brev; however a few of the acoustic characters are ambivalent or even closer to petr than brev. Such an exception is the short IED_S_L_ of lito (Fig 5D–5F). With respect to the syllable duration, hipp is significantly faster than brev (GLM, t_temp_ (1, 25) = -4.3, p_temp_ < 0.001, t_OTUs_ (1, 25) = 2.9, p_OTUs_ = 0.007), but cannot be distinguished from petr (GLM, t_temp_ (1, 27) = -3.9, p_temp_ < 0.001, t_OTUs_ (1, 27) = 0.4, p_OTUs_ = 0.660). The within-lito variation is relatively high between the four song samples. Therefore, in the future, some values should be consolidated with more recordings taken at different ambient temperatures. We regard four individuals from zones of potential contacts between OTUs as “hybrids” (populations Travesio “TR”, Monti Lentini “ML” (2 individuals), Campotosto “CT”).

**Fig 6:**
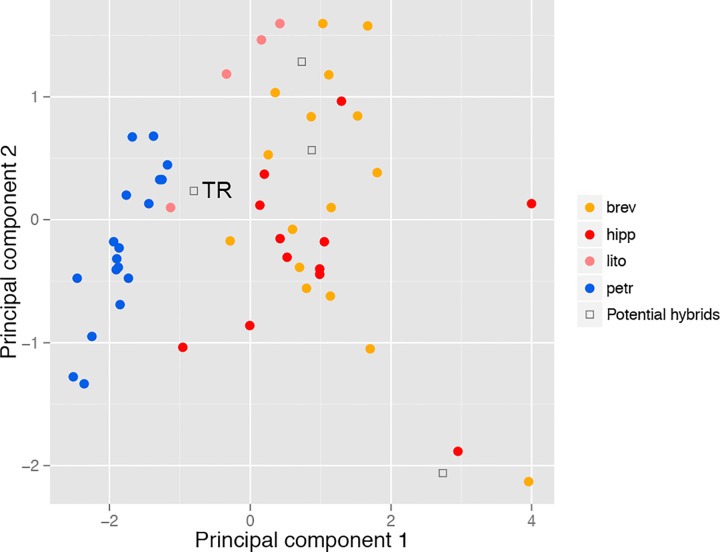
Principal Component Analyses (PCA) among specimens for the three song characters from Fig 5. Specimen mentioned in the text: TR = Travesio population.

Differences are generally small and it is not surprising that a few individuals fall into the wrong OTU when single characters are examined. However, a combination of the three characters from Fig 5 clearly separates petr from the remaining OTUs, especially brev, in a Principal Component Analysis (PCA, Fig 6). A few new recordings from remote Serbian (brev) and Spanish populations (petr) were used to roughly test the acoustic traits.

### Distribution and ecological traits

**Fig 7:**
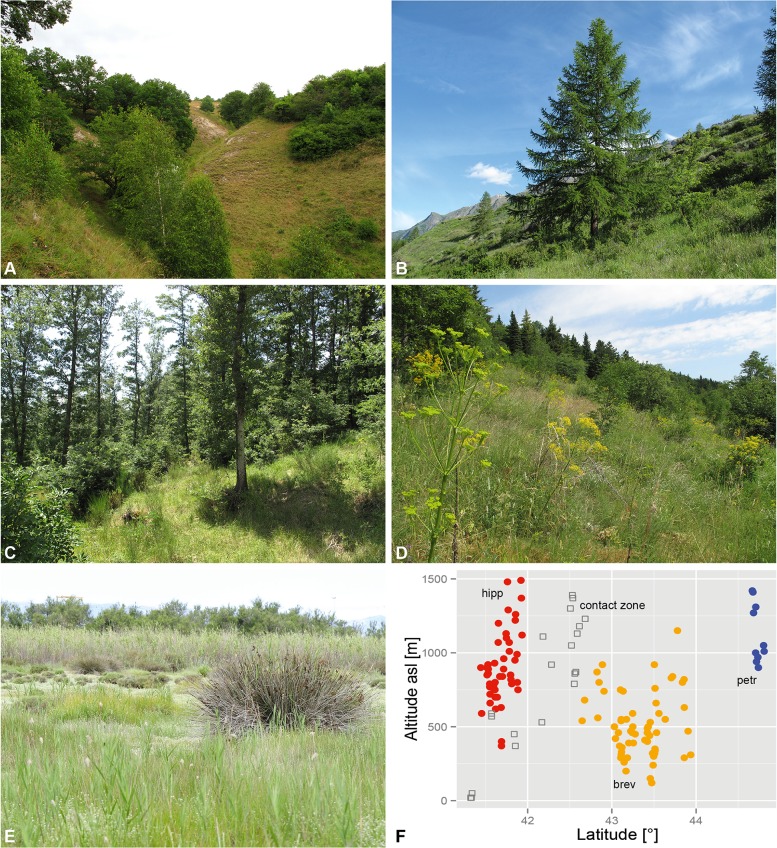
Contrasting habitats in the *Cicadetta brevipennis* group. (A) Dry pastures in the Kyffhäuser (Thuringia, Germany), habitat of petr (locus typicus of *C*. *montana* f. *petryi* Schumacher, 1924); (B) extensive pastures with the European Larch (*Larix decidua*) at 1500 m above sea level (asl) in a south alpine valley (Val de Cogne, Aosta, Italy), extraordinary habitat of petr; (C) oak forests of the foothills (Monte Amiata, Tuscany, Italy), typical habitat of brev in the Apennine Mountains; (D) extensive pastures at higher altitudes in the Monti Della Daunia massif (Apulia, Italy), typical habitat of hipp; (E) salt marsh at sea level at Torreilles (Eastern Pyrenees, France), extraordinary but typical habitat of lito; and (F) altitudinal niches of petr, brev and hipp in the Apennine Mountains visualized against the geographical latitude.

The geographical patterns of the different OTUs are unusual but spatially coherent with the exception of southern French lito near Perpignan (e.g. Figs 3–5). Endemic lito is within the brev-hipp clade in the molecular study but isolated over more than 600 km from its nearest relatives. The acoustic investigations support its closeness to brev and especially hipp. We only know two local lito populations and they live in habitats extraordinary for European cicadas in general and particularly for species of the *C*. *montana* complex: salt marshes at sea level composed of the glasswort (*Salicornia europaea*, Fig 7E).

The OTU we designate as brev occurs in two metapopulations. The Apenninian metapopulation reaches from a northern imaginary line between Pisa (Tuscany) and Forlì (Emilia-Romagna) to a southern line between Terni (Umbria) and Ascoli Piceno (the Marches). Habitats are mainly oak forests in a hilly territory (Fig 7C). Population densities are fairly high for the woody landscapes west of Siena and the main ridge of the Apennine Mountains. The second metapopulation is in Slovenia and southern Austria and probably extends to north-eastern Italy and northern Serbia. We found it here usually on trees and shrubs not far from dry and extensive meadows or other open spaces. The two metapopulations are isolated (over 200 km apart) by the Padan Plain and the Adriatic Sea.

Operational taxonomic unit hipp haplotypes were only found in the Monti Della Daunia population, but we also include in this OTU all populations in the north-west direction at least until the National Parks of Abruzzo and Majella ending in a 120 km to 60 km range (Fig 4B, red line). These populations form a metapopulation that is characterized by mountainous habitats often composed as ecotones with extensive grazed pastures and a high number of bushes (Fig 7D). Operational taxonomic unit hipp inhabits a more mountainous ecological niche than brev (Wilcoxon rank sum test for altitude of all records: W = 351.5, p < 0.001; see also Fig 7F). A few populations between the Apennine brev and hipp metapopulations were not assigned to one of the two groups because they have intermediate song patterns. Some isolated populations at lower altitudes (e.g. Monti Lepini “ML”; Fig 4B) are in the geographic vicinity of hipp but probably are ecologically and acoustically closer to brev or intermediates.

Operational taxonomic unit petr populations are distributed from central Germany (Kyffhäuser, Fig 7A) to the Pyrenees and further to western Spain and along the southern slope of the Alps at least to Lake Como, but also in isolated local populations around Brallo di Pregola (“BR”; e.g. Fig 5) in the northern Apennine entering another mountain chain. In France, petr inhabits Mediterranean and sub-Mediterranean vegetation classes, often composed as ecotones with grassy areas and bushes. Some populations live in forests. North- and eastwards petr is restricted to dry meadows with bushes. Males regularly sing in the herb layer. Exceptional southern alpine habitats were found in the vegetation zone of the European Larch (*Larix decidua*) up to 1750 m above sea level (asl) (Fig 7B).

Populations of petr and brev are separated by no more than approximately 150 km throughout the Apennine Mountains, and likely the southern Alps. However, the single individual investigated acoustically from the south-eastern Italian Alps is intermediate in song patterns (Travesio “TR”; Figs 5 and 6). Operational taxonomic unit bulg was found close to the Black Sea in Bulgaria (Fig 3B).

### Status of operational taxonomic units

Morphological traits (Fig 3C and 3D) and song differences (Figs 1, 5 and 6) are spatially congruent with the petr and brev-hipp-lito clades of the molecular study (Fig 3A and 3B). We thus conclude that the OTU petr is evolving as a separate metapopulation lineage. In contrast, the data suggest that brev and hipp are not evolving separately. We interpret them as two subspecies exhibiting a larger Apenninian contact zone (compare Figs 3B and 4B). The data suggest that lito is a third subspecies. A summary of the taxonomic relevant traits is given in Table 4. We formally describe these taxa later in this paper as follows: petr = *Cicadetta petryi* Schumacher, 1924 described as a form and now regarded as a proper species, brev = *Cicadetta brevipennis brevipennis* Fieber, 1876, hipp = *Cicadetta brevipennis hippolaidica* Hertach ssp. n., lito = *Cicadetta brevipennis litoralis* Puissant & Hertach ssp. n.

**Table 4:** Matrix of taxonomically relevant traits in the *Cicadetta brevipennis* song group.

Character	Data source	petr	brev	hipp	lito
Genetic distance of COI < 1.5% and COII < 1% to *C*. *cerdaniensis*	Genetics	++	– –	– –	– –
E_L_/E_S_ followed by phrase 2: rate < 0.03 (population level)	Acoustics	++	++	– –	– –
Power level EP_L/1_ > 20% of the total power increase	Acoustics	+	–	–	?
IED_S_L_ minimum < 0.34 s	Acoustics	++	– –	– –	++
E_IN_ number < 2.5	Acoustics	+	–	– –	– –
Basal junction of anal veins of fore wing light	Morphology	+	– –	–	++
Veins of apical cell 8 lateral and frontal predominantly dark	Morphology	+	+	++	–
Postclypeus without yellowish spot on the top of the groove	Morphology	+	+	+	–
Habitats at altitudes > 700–800 m asl	Ecology	+/–	–	+	– –
Habitat not in salt marshes	Ecology	++	++	++	– –

Tolerated failure rates < 10% per trait, double symbols for flawless characters in our dataset. For song variables see Figs 1 and 2 and Table 2. Petr = *Cicadetta petryi*, brev = *Cicadetta b*. *brevipennis*, hipp = *Cicadetta b*. *hippolaidica* ssp. n., lito = *Cicadetta b*. *litoralis* ssp. n.

**Fig 8:**
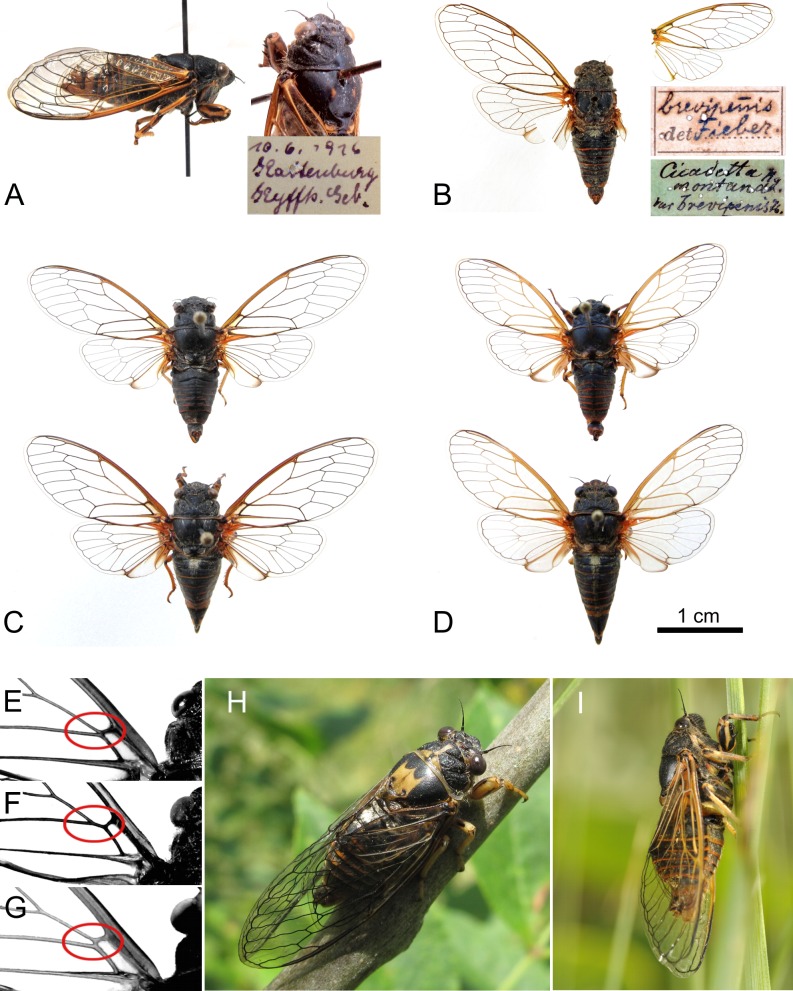
*Cicadetta brevipennis* group morphology. (A) *Cicadetta petryi* holotype in lateral and dorsolateral view from the Natural History Museum of Berlin with original label; (B) *Cicadetta montana* var. *brevipennis* Fieber, 1876 putative type from the Museum of Natural History Vienna with broken wings and original labels; (C) *Cicadetta brevipennis hippolaidica* ssp. n. with holotype specimen (above) and female paratype (below); and (D) *Cicadetta brevipennis litoralis* ssp. n. with holotype specimen (above) and female paratype (below). (E) Normal, (F) aberrant fused and (G) aberrant shifted compositions of median and cubitus anterior veins arising from the basal cell. (H) *Cicadetta b*. *hippolaidica* ssp. n. light morph male and (I) singing *Cicadetta b*. *litoralis* ssp. n in natural conditions.

Schumacher [62] described *Cicadetta montana* f. *petryi* as a distinct colour-morph with a precise type locality in the Kyffhäuser (Germany; “steile Gipshänge der Kattenburg bei Frankenhausen” = steep gypseous slopes at Kattenburg near Frankenhausen). The description was not commented upon until Boulard & Mondon [63] gave species status for specimens from France coloured with four lighter spots on the mesonotum and light basal fore wing venations. Boulard [64] described the calling song of these specimens in the *brevipennis* group scheme as having very long lasting E_L_. Subsequent authors reduced the status of the taxa again to subspecific level or synonymized them completely with *C*. *montana* s. l. (e.g. [47]) or *C*. *brevipennis* (e.g. [38]). These taxonomic decisions were justified insofar as the acoustic and morphological characters given by Boulard were not constant. Schumacher does not provide any song data, but Meineke [65] demonstrated with sonograms that the Kyffhäuser population belongs to the *brevipennis* group. We visited the Kyffhäuser population and identified the characters within the petr species (“KY”; Figs 3–5). Finally, we were able to extract mtDNA of the 100-years-old type specimens preserved in the Natural History Museum of Berlin (“HT”; Figs 3A and 8A). The type specimen is clearly in the petr lineage.

For stability and universality of the nomenclature, we retain the name *Cicadetta brevipennis*. However, in our opinion *Cicadetta montana* var. *brevipennis* sensu Fieber [66] cannot be identified as a song-defined species (see S1 Text). We argue that a putative type specimen (Fig 8B and 8F) which was found in the Museum of Natural History Vienna is doubtful (see S1 Text). Thus, we intend to propose to the ICZN that they disqualify the doubtful Vienna specimen as a potential type. We favour designating a song-determined neotype from the population at Glanz an der Weinstrasse (“GL”) which is only 40 km away from Graz (Austria; compare [61]: Art. 75.3.6). Graz is a locus typicus derived from Fieber’s unpublished plates (National Museum of Natural History Paris).

## Taxonomic Descriptions

### *Cicadetta petryi* Schumacher, 1924

#### Diagnosis

Song characterized by a binary pattern, phrase 2 only emitted in courtship behaviour. Long echemes starting without or with only few introductory chirps (E_IN_ number < 2.5), more chirps possible at low temperatures. Power increasing rapidly in the long echemes (EP_L/1_ > 20%, EP_L/2_ > 38%). Echeme intervals short, especially the pause between the short and the next long echeme (IED_S_L_ minimum < 0.34 s).

92.7% of the *Cicadetta petryi* males and 100% of the females (n_ind_ = 72) have a predominantly light (yellowish) basal junction of anal veins, whereas this wing part is dark in 100% of the *Cicadetta b*. *brevipennis* (n_ind_ = 19). The dark phenotype is also predominant in *C*. *sibillae* (92.3%, n_ind_ = 28), *C*. *montana* s. str. (91.5%, n_ind_ = 71) and *C*. *anapaistica* (97.3%, n_ind_ = 38) among closely related or sympatrically occurring species. From *C*. *montana* s. str., many specimens are additionally distinguished by the generally lighter basal venation and particularly by the outer rim of the costa darker than the inner rim and darker than the radial/subcostal veins in the fore wing (99.2% males and 77.3% females versus 10.0% males and 2.4% females).

#### Examined material

We examined 61 males including the holotype and 11 females (S1 Table). We describe here the variation of the species in comparison to the holotype description of Schumacher [62].

Holotype male: Verbatim label information: “10.6.1916/Kattenburg/Kyffh. Geb.”[label rectangular, white, handwritten] and “*Cicadetta montana*/Scop./f. *Petryi*/nov./*” [label rectangular, white, handwritten] and “Berlin” [label rectangular, white, handwritten] (Natural History Museum of Berlin, Fig 8A).

### Morphology

Males’ variability: Schumacher [62] described his colour form mainly based on two yellowish spots on the mesonotum, which he thought to be unique (Fig 8A, dorsolateral view). In fact, a minority of specimens in all Central European *Cicadetta montana* complex taxa exhibits such smaller or larger yellowish spots. According to our examined material, light markings on the mesonotum are completely lacking in 74.3% of *C*. *petryi*. The “powerful yellowish basal venation” [62] gradually leads to much darker phenotypes; approximately 10% have some basal veins dark brown or even black. In the males we examined, the outer rim of the costa is generally darker than the inner rim of costa and darker than the radial/subcostal vein (with one specimen ambivalent), but colour tints are sometimes different from Schumacher, for example brown for the outer rim of costa and ochre for both other rims.

Schumacher pointed out the stocky and short body of his type. *Cicadetta petryi* tends to be a rather small species within the complex, but with high variability (Table 5). However, specimens are not particularly stocky (ratio of body length to width measured at tergite 2: 3.08 ± 0.11, males only), which is obvious in comparison for example with *C*. *anapaistica* (2.99 ± 0.08). The wing ratio (“2.5 to 2.6”; [62]) can be specified to a range from 2.31 to 2.62 (males only).

**Table 5:** Body and wing measurements within the *Cicadetta brevipennis* group.

		*C*. *petryi*	*C*. *b*. *brevipennis*	*C*. *b*. *hippolaidica*	*C*. *b*. *litoralis*
**Males**		**(n**_**ind**_ **= 61)**	**(n**_**ind**_ **= 17)**	**ssp. n. (n**_**ind**_ **= 12)**	**ssp. n. (n**_**ind**_ **= 21)**
Body length [mm]	Mean ± SD	17.2 ± 0.7	17.6 ± 1.0	16.9 ± 0.7	17.8 ± 0.8
	Maximum	19.2	19.5	18.3	19.5
	Minimum	15.7	15.4	15.9	16.1
Body width [mm]*	Mean ± SD	5.6 ± 0.3	5.7 ± 0.2	5.6 ± 0.2	5.7 ± 0.2
	Maximum	6.2	6.2	5.9	6.0
	Minimum	5.0	5.3	5.1	5.3
Fore wing length [mm]	Mean ± SD	18.3 ± 0.8	19.4 ± 0.9	18.7 ± 0.5	18.0 ± 0.8
	Maximum	20.4	21.3	19.4	19.4
	Minimum	16.4	18.0	17.7	16.3
Fore wing width [mm]	Mean ± SD	7.4 ± 0.4	7.7 ± 0.4	7.5 ± 0.3	7.5 ± 0.4
	Maximum	8.5	8.6	8.3	8.2
	Minimum	6.6	7.3	7.1	6.8
**Females**		**(n**_**ind**_ **= 11)**	**(n**_**ind**_ **= 2)**	**(n**_**ind**_ **= 2)**	**(n**_**ind**_ **= 2)**
Body length [mm]	Mean ± SD	18.5 ± 0.7	18.6 ± 0.0	17.7 ± 0.3	20.0 ± 0.6
Body width [mm]*	Mean ± SD	5.8 ± 0.3	5.9 ± 0.1	5.6 ± 0.3	6.1 ± 0.3
Fore wing length [mm]	Mean ± SD	19.7 ± 0.7	20.8 ± 0.5	19.4 ± 0.0	18.3 ± 0.8
Fore wing width [mm]	Mean ± SD	8.1 ± 0.3	8.0 ± 0.4	8.0 ± 0.1	7.9 ± 0.5

*measured at tergite 2 of the abdomen

Females’ variability: Colouration does not differ significantly from the variability given for males. However, their venation tends to be lighter, which results in more specimens having the outer rim of costa not darker than the two neighboured rims.

### *Cicadetta brevipennis brevipennis* Fieber, 1876

#### Diagnosis

Song characterized by a binary pattern, phrase 2 only emitted in courtship behaviour. Long echemes starting with a series of introductory chirps (E_IN_ number > 2.5). Power increasing slowly in the long echemes (EP_L/1_ < 20%, EP_L/2_ < 38%). Echeme intervals long, especially the pause between the short and the next long echeme (IED_S_L_ minimum > 0.34 s).

*Cicadetta b*. *brevipennis* can be separated with high probability by the predominantly dark basal junction of the anal veins (100%, n_ind_ = 19) from *C*. *petryi* and *C*. *cerdaniensis* (5%, n_ind_ = 20). Again, many specimens are distinguished from *C*. *montana* s. str. by the outer rim of costa darker than the inner rim and darker than the radial/subcostal veins in the fore wing (89.5% versus 9.5%).

#### Examined material

We describe the variation of 19 specimens, 17 males and 2 females (S1 Table) in comparison to Fieber [66].

### Morphology

Males’ variability: Fieber’s [66] *C*. *montana* belongs to the taxa with basal part of the fore wing venations predominantly brownish or yellowish which fits with our material. He describes the varieties *C*. *montana* var. *brevipennis* and *C*. *montana* var. *longipennis* with a relatively large number of characters. While a few of the characters are truly ambiguous, others are dependent on the preparation or on the age of the specimen. We omit such characters and concentrate on the remaining ones (“[given in quotation marks]”) and list them in the order provided by Fieber.

The lateral part of the pronotal collar is often “rounded”, but about one third of our specimens have a straight margin. Its angle is usually short, but we would in most specimens not have called the end “almost truncated” as Fieber did. The groove of the clypeus is normally “narrow”; sometimes parts of it are enlarged. The margins of the fore wing are rarely almost “monochrome yellow to red”, but normally trichromatic with the outer rim of costal vein darker than the inner rim and darker than the subcostal/radial vein. All specimens have a dark junction of the anal veins, which unfortunately is not a character listed by Fieber. The big majority of specimens are with genus-typical origin of median and cubitus anterior veins at one point at basal cell (Fig 8E; but compare S1 Text). The yellowish part of the operculum covers, in all specimens of our sample, the apical half rather than “only the apical margin”. This apical margin is normally and in accordance with Fieber “not recessed”. The meracanthus is often slightly bent sidewise and straight, but only rarely “curved and hooked”; and only the base of the meracanthus is “blackish”, but the tip is much lighter. The median lobe of the uncus is variable in shape and length; often it is “short”, sometimes “almost semi-circular”. Sternites III to V frequently possess “dark spots”. These spots have variable, rarely “semi-circular” shapes but are by trend “shrinking”. On sternites VI and VII spots are more often missing than observed by Fieber. The latter is wider than long and, therefore, scarcely “elongated” in the sense of Fieber, but normally “trapezoidal”. Sternite VIII is in fact “elongated and oval, narrower towards the tip”. A minority has–disagreeing with Fieber–a faint central ridge. For body and wing sizes see Table 5.

Females’ variability: The two females do not differ significantly from the variability given for males. Their frontal margins of the fore wings tend to exhibit less pronounced colour tints.

### *Cicadetta brevipennis hippolaidica* Hertach ssp. n.

see urn:lsid:zoobank.org:act:AE8F67BE-A904-491F-BE7E-5EA7FDF39900

#### Diagnosis

Song characterized by a binary pattern, phrase 2 is regularly or at least from time to time emitted in the calling song (frequency of occurrence > 0.03 on the population level) and relatively loud. Long echemes starting with a series of introductory chirps (E_IN_ number > 2.5). Power increasing slowly in the long echemes (EP_L/1_ < 20%, EP_L/2_ < 38%). Echeme intervals long, especially the pause between the short and the next long echeme (IED_S_L_ minimum > 0.34 s).

*Cicadetta brevipennis hippolaidica* ssp. n. occurs in two distinct morphs. The dark coloured morph resembles all other described *C*. *montana* complex species. However, the vast majority of *Cicadetta b*. *hippolaidica* ssp. n. specimens, like *C*. *b*. *brevipennis*, are separated from *C*. *petryi* by the predominantly dark basal junction of anal veins (95.5% for *C*. *b*. *hippolaidica* ssp. n. (dark morphs only), 89.3% for *C*. *b*. *hippolaidica* ssp. n. (dark and light morphs) vs. 6.2% for *C*. *petryi*). From *C*. *montana* s. str., many specimens are distinguished by the outer rim of costa darker than the inner rim and darker than the radial/subcostal veins (92.9% vs. 9.5%). Light coloured morphs are clearly distinguished by large ochre markings on the pronotum and mesonotum from all other described species with the exception of some *C*. *anapaistica lucana* Hertach, 2015, a taxon exhibiting the same dimorphism [2], and some southern French populations of *C*. *petryi* approaching typical light morphs.

#### Type series

The type series consists of 12 males and two females representing the whole distribution range but with a clear focus on the Monti Della Daunia population (6 types). The specimens are kept in the Natural History Museum of Basel (NHMB), the Natural History Museum of Bern (NMBE) and one private collection.

Holotype male: Verbatim label information: “Monte Sambuco, PUGL, I/41.5311°/15.0825°, 890 m asl/28.6.2011, leg. Thomas Hertach” [label rectangular, white, printed] and “HOLOTYPUS ♂/*Cicadetta brevipennis hippolaidica* ssp. n./Hertach 2016” [label rectangular, light red with dark red margin, printed] (NHMB).

Paratypes: All paratypes with labels “PARATYPUS XX Y, *Cicadetta brevipennis hippolaidica* ssp. n. Hertach 2016” [label rectangular, white with red margin, printed] at which “XX” is the number of the paratype and “Y” the sex of the specimen. Number “2” does not exist. Paratypes males, dark morph: Monte Sambuco, Monti della Daunia, APUL, I, 41.5312°/15.0830°, 860 m asl, 19.7.2010, leg. T. Hertach (paratype 1, coll. Hertach); Monte Sambuco, Monti della Daunia, APUL, I, 41.5311°/15.0825°, 890 m asl, 28.6.2011, leg. T. Hertach (paratype 4, coll. Hertach); N Torrebruna, ABRU, I, 41.8846°/14.5297°, 750 m asl, 1.7.2011, leg. T. Hertach (paratypes 7 to 9, coll. Hertach); S Pietrabbondante, MOLI, I, 41.7158°/14.3767°, 960 m asl, 2.7.2011, leg. T. Hertach (paratype 10, coll. NMBE); Lago Selva, Cardito, LAZI, I, 41.6071°/13.9758°, 930 m asl, 3.7.2011, leg. T. Hertach (paratype 11, coll. Hertach); Picinisco-Mainarde, LAZI, I, 41.6527°/13.9037°, 930 m asl, 3.7.2011, leg. T. Hertach (paratype 12, coll. Hertach). Paratypes males, light morph: Monte Sambuco, Monti della Daunia, APUL, I, 41.5311°/15.0825°, 890 m asl, 28.6.2011, leg. T. Hertach (paratype 3, NMBE); Monte Sambuco, Monti della Daunia, APUL, I, 41.5284°/15.0838°, 920 m asl, 28.6.2011, leg. T. Hertach (paratype 5, coll. Hertach); Monte Sambuco, Monti della Daunia, APUL, I, 41.5490°/15.1036°, 790 m asl, 29.6.2011, leg. T. Hertach (paratype 6, coll. Hertach). Paratypes females: N Pescasseroli, ABRU, I, 41.8564°/13.7847°, 1260 m asl, 4.7.2011, leg. T. Hertach (paratype 13, coll. NMBE); N Pescasseroli, ABRU, I, 41.8564°/13.7847°, 1260 m asl, 4.7.2011, leg. T. Hertach (paratype 14, coll. Hertach).

#### Morphological description

The frequency of the two different coloured morphs in *C*. *b*. *hippolaidica* ssp. n. is difficult to estimate over the whole distribution range. Light morphs were so far only caught in the Monti Della Daunia population with a probability of 50%.

Male holotype with remarks on the variability of dark morph paratypes (Fig 8C): Body length: 17.2 mm, body width (tergite 2): 5.7 mm, fore wing length: 19.2 mm, fore wing width: 7.6 mm (for variability in size of the type series see Table 5).

Head: Black with ochre patch on epicranial suture (in paratypes rarely additionally frontal to posterior margin ochre). Postclypeus with longitudinal narrow groove, black with lateral margins ochre (in paratypes rarely with a yellowish triangle patch at postclypeus towards the frontoclypeal suture), anteclypeus predominantly black. Rostrum reaching mid trochanter, labrum ochre, mentum lateral brown, labium blackish. Antennae dark brown to black with lighter margins of scapes.

Thorax: Pronotum generally black (in paratypes sometimes with a light spot on the central fissure), posterior margin of pronotal collar towards the angles ochre, central interrupted (in one paratype completely black, in others without interruption). Lateral angles of pronotal collar pronounced, pronotal collar frontal with turned up margin and straight in dorsal view (in paratypes sometimes convex in shape). Scutum, cruciform elevation and metanotum black, the latter with yellowish margins (in paratypes rarely with paired lighter spots on the cruciform elevation or on the scutum). Ventral side generally black, except membranes at bases of legs orange to brown. Opercula not overlapping, kidney-shaped with black base and ochre distal part. Meracanthus with straight spike directed slightly laterally (in paratypes rarely bent inward).

Abdomen: Abdomen triangular in cross section. Tergites I and II black (in paratypes rarely tergite II with lighter sections), tergites III through VII frontal black and caudal with small orange to brown bands strongly narrowed to dorsal ridge, tergite VIII light portions more important. Sternite VIII large and long. Sternites III through VII brown with darker sectors lessened towards the end of abdomen (in paratypes rarely spots missing or cloudy). Epipleurites caudally brown, frontally darker. Timbals with three ribs, two long and one shortened, and timbal plate.

Legs: All legs with yellowish and black fasciae and dots, mid and hind tibiae predominantly yellow. Fore femurs holding three spines with decreasing length towards the tibia.

Wings: Fore wing hyaline except for slightly yellowish basal cell and for brownish pterostigma. Basal membrane orange. Median and cubitus anterior vein originating in one point at basal cell (in some paratypes with median and cubitus anterior vein fused for less than 1 mm on one or both sides; Fig 8E and 8F). Colouration of basal veins ranging from ochre to dark brown. Exterior rim of costal vein darker than inner rim and radial/subcostal vein (in paratypes rarely all three rims light). Basal junction of anal veins predominantly dark brown to black (Figs 3C and 8). Distal veins almost black with eight apical cells (in two paratypes with one-sided seven cells). Hind wing transparent except for orange base of costal cell, orange to brownish margins of jugum and plaga and dark brown spotted apical vannus margin. Veins dark, especially in distal part, with six apical cells. Cubitus anterior vein lighter than median vein.

Genitalia: Pygofer predominantly dark with lateral parts ochre with rounded dorsal beak and blunt upper lobes. Median lobe of uncus blackish and curved upwards (in some paratypes with yellowish parts), tongue-shaped and rather broad. Claspers hooked and dark brown. Pseudoparameres flattened and with sharp end. Anal tube and anal style reddish to brown orange.

Male paratypes of light morph (Fig 8H): Contrary to the dark morph several parts of the body are coloured ochre or almost golden instead of black. On the head, postclypeus towards the frontoclypeal suture and sometimes towards the anteclypeus, the anteclypeus itself as well as the surrounding of the compound eyes ochre. On the pronotum, central suture, frontal margin and pronotal collar appearing as broad ochre bands. On the mesonotum, completely ochre except for the submedian and lateral sigillae and the scutal depressions (in two paratypes) or additionally except for the central part (in one paratype). Cruciform elevation and its lateral depressions completely ochre. Ventral side of thorax in one paratype significantly lighter. At the legs, light portions more dominant, especially at the fore leg. One paratype with basal junction of anal veins ochre instead of dark at fore wing.

Female paratypes (Fig 8C): Both females are perfectly within the variation of the dark morph males. On the pronotum, one female with a yellowish spot on the central fissure, the other with a continuous light posterior margin. Scutum and cruciform elevations completely black. On fore wings, the basal veins relatively light, but still exterior rim of costal vein darker than inner rim and radial/subcostal vein, and basal junction of anal veins dark. Genitalia: Tergite IX dorsal dark abruptly narrowed halfway, lateral brown red to golden. Dorsal beak rounded, anal styles orange. Ovipositor brown orange, tip dark. Ratio of body length to ovipositor length (including sheath) 2.8 and 2.6.

#### Etymology

The new subspecies *C*. *b*. *hippolaidica* ssp. n. is characterized by regular repetitions of phrase 2 in the calling song. This element is close to the song pattern (type 1; [30,67]) of the tiny cicada *Tettigettula pygmea* (Olivier, 1790). The similarity is so striking (Fig 1G and 1H) that it seems as if *C*. *b*. *hippolaidica* ssp. n. sometimes imitates the numerically dominant syntopic *T*. *pygmea*. This imitation is unlikely to be real because cicadas are not expected to learn songs from other individuals or species [32]. Nevertheless, we use this amusing phenomenon to name the new subspecies after the tree warbler bird genus *Hippolais* von Baldenstein, 1827 where different European species are excellent imitators of non-conspecific songs.

### *Cicadetta brevipennis litoralis* Puissant & Hertach ssp. n.

see urn:lsid:zoobank.org:act:E00152C3-8113-4BBC-8D7C-E20D0F980699

#### Diagnosis

Song characterized by a binary pattern, phrase 2 is regularly emitted in the calling song (frequency of occurrence >> 0.03 on population level). Long echemes starting with a series of introductory chirps (E_IN_ number > 2.5). Power increasing rather slowly in the long echeme. The pause between the short and the next long echeme fast (IED_S_L_ minimum < 0.34 s). Long echeme durations highly variable.

*Cicadetta brevipennis litoralis* ssp. n. is the taxon within the *C*. *montana* species complex with the lowest forewing length to body length ratio and the lightest colour venations known (Fig 8D and 8I). A high number of specimens can be distinguished from the other taxa described from Western, Central and Southern Europe. Central hind wing venations are predominantly coloured light brown, yellow, ochre or whitish (93.5%, n_ind_ = 23) whereas the majority of venations are darker (brown or black) in all other taxa (98.9%, n_ind_ = 315). Apical cell 8 (without ambient venation) is surrounded by predominantly light brown, yellow or ochre venations (91.3%) against predominantly darker colouration (close to the tint of the ambient vein) for the other taxa (98.1%). The constantly ochre or yellowish colouration of the top of the postclypeal groove (95.6%) is unusual in comparison to the other *brevipennis* group taxa (3.2%) as is the yellow-tinting of the wings. Fore wing length to body length ratio in males is 1.01 ± 0.04 (min 0.91, max 1.11) but for example in *C*. *petryi* 1.07 ± 0.04, in *C*. *b*. *brevipennis* 1.11 ± 0.06 and in *C*. *b*. *hippolaidica* ssp. n. 1.11 ± 0.03. Contrary to *C*. *b*. *brevipennis* and *C*. *b*. *hippolaidica* ssp. n., the basal junction of anal veins is yellowish (100%) as in *C*. *petryi*.

#### Type series

The type series consists of 21 males and two females representing the two known local populations near Perpignan. It is kept in the National Museum of Natural History Paris (MNHN), the Slovenian Museum of Natural History Ljubljana (PMSL) and two private collections. For nature conservation reasons we omit verbatim label information and rounded the geographic coordinates.

Holotype male: Torreilles, Eastern Pyrenees, F, 42.76°/3.02°, 1 m asl, 19.5.2001, leg. S. Puissant and “*Cicadetta montana*/Dét. S. PUISSANT 2001” [three labels rectangular, white, hand-written] and “HOLOTYPUS ♂/ *Cicadetta brevipennis litoralis* ssp. n./Puissant & Hertach 2016” [label rectangular, light red with dark red margin, printed] (MNHN).

Paratypes: All paratypes with labels “PARATYPUS XX Y, *Cicadetta brevipennis litoralis* ssp. n. Puissant & Hertach 2016” [label rectangular, white with red margin, printed] at which “XX” is the number of the paratype and “Y” the sex of the specimen. Paratype males: Alénya, Eastern Pyrenees, F, 42.65°/2.99°, 5 m asl, 31.5.1999, leg. S. Puissant (paratypes 1 and 2, coll. Puissant); Torreilles, Eastern Pyrenees, F, 42.76°/3.02°, 1 m asl, 31.5.1999, leg. S. Puissant (paratype 3, coll. Puissant); Torreilles, Eastern Pyrenees, F, 42.76°/3.02°, 1 m asl, 5.6.1999, leg. S. Puissant (paratype 4, coll. Puissant); Torreilles, Eastern Pyrenees, F, 42.76°/3.02°, 1 m asl, 19.6.1999, leg. S. Puissant (paratypes 5 and 6, coll. Puissant); Torreilles, Eastern Pyrenees, F, 42.76°/3.02°, 1 m asl, 19.5.2001, leg. S. Puissant (paratypes 7 and 8, coll. Puissant); Torreilles, Eastern Pyrenees, F, 42.76°/3.02°, 1 m asl, 24.5.2001, leg. S. Puissant (paratypes 9 and 10, coll. Puissant); Torreilles, Eastern Pyrenees, F, 42.76°/3.02°, 1 m asl, 14.6.2002, leg. S. Puissant (paratypes 13 to 20, coll. PMSL); Torreilles, Eastern Pyrenees, F, 42.76°/3.02°, 1 m asl, 4.6.2013, leg. S. Puissant (paratypes 21 and 22, coll. Hertach). Paratype females: Torreilles, Eastern Pyrenees, F, 42.76°/3.02°, 1 m asl, 24.5.2001, leg. S. Puissant (paratype 11, coll. MNHN); Torreilles, Eastern Pyrenees, F, 42.76°/3.02°, 1 m asl, 26.5.2001, leg. S. Puissant (paratype 12, coll. Puissant).

### Morphological description

Male holotype (Fig 8D): Body length: 16.8 mm, body width (tergite 2): 5.4 mm, fore wing length: 17.5 mm, fore wing width: 7.4 mm (for variability in size of the type series see Table 5).

The holotype specimen of *Cicadetta brevipennis litoralis* ssp. n. fits the detailed description of the holotype of *C*. *b*. *hippolaidica* ssp. n. with the following differences: On the head, longitudinal narrow groove of the postclypeus on the top ochre. Supra-antennal plate partly ochre. On the thorax, posterior margin of pronotal collar ochre without central interruption, frontal to the angles convex and clearly recessed in dorsal view (as in some paratypes of *C*. *b*. *hippolaidica* ssp. n.). Cruciform elevation brownish, its lateral depressions ochre (as in some light morph paratypes of *C*. *b*. *hippolaidica* ssp. n.). Posterior margins of mesonotum and metanotum remarkably light. On the abdomen, sternite VII without darker spot (as in some paratypes of *C*. *b*. *hippolaidica* ssp. n.). Wings appear tinted yellowish and not completely colourless. On the fore wings, median and cubitus anterior vein originating at two slightly shifted points at basal cell (Fig 8G). Basal venation remarkably yellow to whitish ochre including the basal cell and the basal junction of anal veins but with a darker exterior rim of costa and posterior margin with anal veins 2 and 3. Distal veins still light with the exception of the ambient venation and adjoining bases of veins leading away. In particular, apical cells lateral and frontal predominantly light bordered. On the hind wings, the ambient, the cubitus posterior and the anal 2 veins are dark while the remaining veins are very light yellow to whitish ochre.

Male paratypes: Male paratypes of *C*. *b*. *litoralis* ssp. n. differ from the holotype as follows: On the thorax, light colouration at posterior margin of pronotal collar sometimes interrupted, more rarely completely missing (both variants as in some *C*. *b*. *hippolaidica* ssp. n.). Frontal margin often also marked lighter, in one paratype with spot on central fissure. Approximately half of the paratypes with more or less straight lateral margins of pronotal collar in dorsal view (as in some *C*. *b*. *hippolaidica* ssp. n.). Cruciform elevation often with lighter paired spots, rarely completely light or completely dark. Scutum occasionally with two small spots (as in some *C*. *b*. *hippolaidica* ssp. n.). Meracanthus sometimes bent inward (as in some paratypes of *C*. *b*. *hippolaidica* ssp. n.). On the abdomen, sternites normally with well pronounced dark spots also on segment VII (as in some *C*. *b*. *hippolaidica* ssp. n.). On the fore wings, specimens with genus-typical origin of median and cubitus anterior veins at one point at basal cell are outnumbered against the formation described for the holotype (estimated ratio 1:2). A few paratypes with a short fusion of median and cubitus anterior veins at the base (as in a minority of *C*. *b*. *hippolaidica* ssp. n.; Fig 8E–8G). Colouration of the veins also in paratypes light and rather constant. Apical cells lateral or/and frontal rarely darker towards the colour tint of the ambient vein. Hind wing rarely predominantly darker veined, but cubital anterior veins light in all specimens.

Female paratypes (Fig 8D): Both females are similar to the majority of males with respect to colouration of body and wings. On the fore wings, one female with median and cubitus anterior vein originating at two slightly shifted points at basal cell, the other with shortly fused alternative. Genitalia: Tergite IX dorsal dark, lateral brown red, dark colouration continuously narrowed to the end. Dorsal beak rounded, anal styles orange. Ovipositor brown orange, tip dark. Both females with long body, but short wings: ratio wing length to body length 0.90 and 0.92 (1.07 ± 0.04 for other *brevipennis* group taxa pooled). Ratio of body length to ovipositor length (including sheath) 3.0 for both specimens.

#### Etymology

*Cicadetta b*. *litoralis* ssp. n. lives in extraordinary habitats influenced by salt-water near the coast (Figs 7E and 8I). “*Litoralis*” is Latin adjective meaning “coastal” or “belonging to the coast”.

## Discussion

### Parallels between the *Cicadetta cerdaniensis* and *Cicadetta brevipennis* song groups

**Fig 9:**
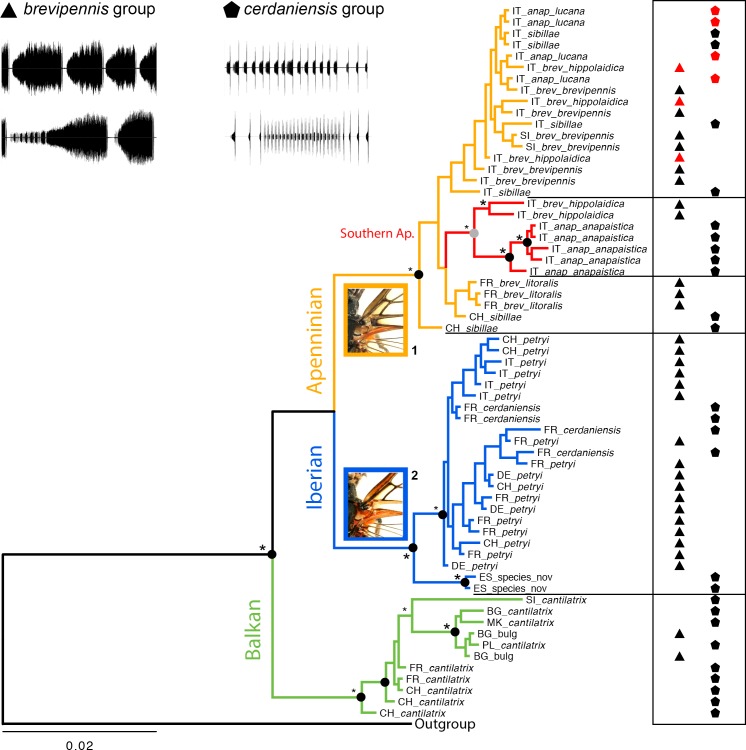
COI and COII concatenated phylogeny of the *Cicadetta brevipennis* and *Cicadetta cerdaniensis* song groups. Data set expanding previous studies [2,3] with eight newly sampled specimens. Bayesian phylogeny showing hypothesized geographic origins of the clades, the song groups of individual specimens and a colouration trait. Notes: Bayesian posterior probabilities (filled circles: black > 0.95, grey > 0.90) and ML bootstrap values (star icons: large > 0.90, small > 0.80) from RAxML analysis (S3 Fig). Red song-symbols identify specimens with southern Apenninian origin presumably introgressed by central Apenninian taxa. Country codes: BG = Bulgaria, CH = Switzerland, DE = Germany, ES = Spain, FR = France, IT = Italy, MK = Macedonia, PL = Poland, SI = Slovenia. OTU bulg is poorly known. Basic and complicated acoustic examples of both song groups (15 s; *Cicadetta petryi*, top left; *Cicadetta brevipennis hippolaidica* ssp. n., below left; *Cicadetta cantilatrix*, top right; *Cicadetta anapaistica anapaistica*, below right). ^1^Colouration trait not valid for endemic *Cicadetta brevipennis litoralis* ssp. n., ^2^Spanish undescribed taxon ambivalent dark or light.

Our work has revealed astonishing parallels between the *cerdaniensis* and the *brevipennis* song groups for different character sets. The most striking result is that the mtDNA identified four inter-group species pairs (Fig 9). Similar haplotypes are not only present in the pairs *C*. *petryi*/*C*. *cerdaniensis* s. str. (blue; former A-lineages) and *C*. *b*. *brevipennis*/*C*. *sibillae* (orange; former B-lineages; [2,3]) but also in bulg and *C*. *cantilatrix* (green). Moreover, the sister taxon of one *C*. *b*. *hippolaidica* ssp. n. haplogroup is *C*. *a*. *anapaistica* (red) another taxon of the *cerdaniensis* song group. In the *brevipennis* group as well as in the *cerdaniensis* group central and southern Apenninian (Italy) taxa share mtDNA haplotypes.

Another similarity arises from wing colouration. Yellowish or dark basal junctions of anal veins are not only indicative for the *brevipennis* song group but also for approximately 95% of the specimens to distinguish between *C*. *cerdaniensis* s. str. on the one hand (yellowish) and *Cicadetta sibillae* and *Cicadetta anapaistica* on the other hand (dark) in the *cerdaniensis* song group [2]. Thus, Apenninian taxa of both song groups tend to have dark basal junctions of anal veins, while western taxa are normally yellowish (Figs 3C, 3D and 9). Much lighter coloured morphs occur in southern Apenninian taxa of both song groups, in *C*. *b*. *hippolaidica* ssp. n. (Fig 8H) and *C*. *a*. *lucana*; these taxa are separated maximally by 65 km at present.

Even in the clearly distinct song patterns, we can find similarities between the two groups. The southern Apenninian calling song patterns are the most complex in rhythm (*C*. *anapaistica* and *C*. *b*. *hippolaidica* ssp. n.), while the western and the central Apenninian species (*C*. *cerdaniensis* versus *C*. *sibillae* and *C*. *petryi* versus *C*. *b*. *brevipennis*) can mainly be separated by quantitative and scarcely by qualitative characters. The central Apenninian taxa (*C*. *sibillae* and *C*. *b*. *brevipennis*) move their timbals slower than western and southern sister taxa (compare [2]). The sum of the parallel traits is so striking, that it could help to better understand the biogeography and phylogeny of both groups.

Moreover, *Cicadetta b*. *litoralis* ssp. n. and *C*. *b*. *hippolaidica* ssp. n. have evolved phrase 2 to function differently from other taxa (i.e. as a calling song element rather than a courtship element). A similar case is documented for the *cerdaniensis* group where *C*. *sibillae* and *C*. *cerdaniensis* emit a phrase in the calling song that is known only as part of the courtship behaviour in *C*. *cantilatrix* [2].

### Integrative species delimitation and the meaning of “species”

**Fig 10:**
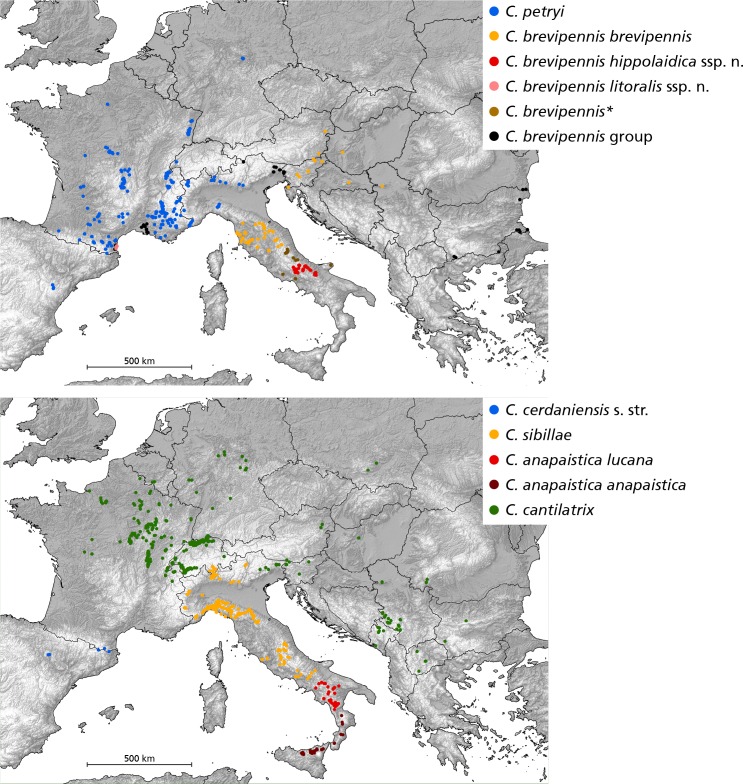
Distribution maps of *Cicadetta brevipennis* song-group taxa (top) and *Cicadetta cerdaniensis* song-group taxa (below). Many records in our database were sampled prior to our recent studies and do not contain sufficient audio information. In these cases taxon assignment to the various *Cicadetta brevipennis* group taxa is based on biogeography and must be verified especially for black dotted populations. Some of the records were previously published, essentially records of the *cerdaniensis* group [2,38–42,65,71–79]. *Brown dots are observations of *Cicadetta brevipennis* without assignment to a subspecies in Central Italy. Some of these populations mark hybrids between *C*. *b*. *brevipennis* and *C*. *b*. *hippolaidica* ssp. n.

The failure to recover the *cerdaniensis* group and the *brevipennis* group as two reciprocally monophyletic clades was surprising, especially to the bioacousticians. Within-song-group patterns are so closely related and among-group differences so complex that these groups should each have one common ancestor; one for the *cerdaniensis* group and one for the *brevipennis* group. In the absence of song, systematists would have concluded the existence of a western, a north-eastern and an Italian taxon, respectively, some supported by colouration traits (Fig 9). Phylogeny-based species delimitation including both mitochondrial and nuclear genes *elongation factor 1-alpha* and *period* using the GMYC method recovered these three geographic entities [3]. The same result emerged when working with multiple user-provided guides in the Bayesian coalescent species delimitation program BPP [3]. This kind of delimitation is very strange in repeatedly combining populations that have such distinct songs. A naive observer might describe each mtDNA clade as displaying a song-polymorphism, but the consideration of the combined datasets as a whole plus studies of many other cicada species complexes, argue against this explanation. Songs are innate in cicadas and decisive for the attraction of conspecific females and have been demonstrated to distinguish cryptic species of cicadas (e.g. [30,32,68]) and many other insect taxa. The *brevipennis* and *cerdaniensis* groups must be regarded as stable units. Among thousands of individuals, we recorded or heard, at most, three times, mixed-group song fragments (always in syntopic populations between *C*. *sibillae* and *C*. *petryi*). The presence of intermediate song characters in an area of contact is good evidence for hybridization (e.g. [2,32,69]). These observations are hints that recent sexual inter-group contacts exist, but at a very low level. Hybrid songs are normally expected to be unattractive and can be regarded as pre-zygotic behavioural barriers to allospecific females [70]. The inter-group stability can also be demonstrated with respect to the distribution patterns: The *brevipennis* group shows an allopatric to parapatric distribution and the *cerdaniensis* group contains five allopatrically to parapatrically distributed taxa with similar but not exactly the same geographic boundaries (Fig 10; [2]).

Our system illustrates that it is not wise to rely on single or even two standard sources of taxonomic information (mtDNA, colouration) when delimiting species. Many difficult taxa can only be resolved when data from multiple data sources are combined. Songs often evolve faster than morphology and are considered to be the best markers of the early stages of reproductive isolation (e.g. [27,31,32,80]). Cicada complexes with morphologically similar but acoustically different species have repeatedly been shown to exhibit irregular mtDNA phylogenies [32,68,81,82].

In empirical studies most often morphological differences are combined with mitochondrial DNA, either the first are validated with the second or vice versa [21,22]. When these two data sources contradict each other, many authors evaluate results against a nuclear background and (if divergent enough) can demonstrate incomplete lineage sorting or introgression among lineages (e.g. [16,18,83,84]). Our study taxa have the disadvantage, that the nuclear genes examined to date (*elongation factor 1-alpha* and *period*) are not informative for recent cladogenic events, such as those we are studying here, when subjected to a variety of species delimitation methods [3]. The closest model organism to cicadas with a complete genome sequenced is an aphid (Sternorhyncha) and next-generation sequencing is needed to target fast evolving protein-coding genes for cicada taxonomy [45]. Luckily, cicada songs provide an applicable, nuclear-encoded, powerful discrimination tool more relevant to cicada biology than the average nuclear gene.

Different authors recently criticized the statistical rigour and objectivity of species delimitation (e.g. [85,86]). However, it is equally problematic to rely exclusively on generalized species delimitation models that are imperfect imitations of the biological reality, most of them for example not allowing divergence with gene flow or the inclusion of non-molecular data (e.g. [12,13,22]). Our simple but practical guide is to use a combination of song, morphology, mtDNA and distributional data to recognize lines of evidence for “separately evolving metapopulation lineages” [4]. We emphasize however that “separately evolving” does not imply a lack of gene flow between species. De Queiroz [4] qualified his concept by saying that, lineages do not have to be phenetically distinguishable, diagnosable, monophyletic, or intrinsically reproductively isolated. We suggest that De Queiroz’ view of “separately evolving metapopulations”, just as Stebbins [87] idea of species occupying “separate evolutionary trajectories”, are not in conflict with Mallet’s [5] restatement of the Darwinian view that species are one step in a continuum from varieties to full species and that, “the existence of this continuum provides good evidence for gradual evolution of species from ecological races and biotypes, to hybridizing species and, ultimately, to species that no longer cross.” Our data analysis, species definitions, and evolutionary scenarios are all consistent with these ideas. Our results suggest that hybridization can take place between local populations of the *Cicadetta* species studied here within the range suggested by Mallet (< 0.1% per generation; [5]). We designate as “species”, taxa that appear to have little to no gene flow among them as measured by our suite of characters. We designate as “subspecies” taxa that appear to be exchanging genes to a degree that blurs the differences.

We analyse here data from the wide distribution area of *C*. *brevipennis* s. l. where song differences were initially not obvious but became clearer when quantitative song characteristics were measured. Combining small acoustic, morphological and ecological traits with the molecular data we noticed unexpected congruence and spatial coherence among datasets and among OTUs. Phenotypic differences corresponded to genotypic clades, even at localities where biogeographical patterns are unusual. This is especially true for the subspecies *C*. *b*. *litoralis* ssp. n. in southern France and for the Apenninian populations of *C*. *petryi* (Brallo di Pregola “BR”), but also for the disjunct *C*. *b*. *brevipennis* distribution. The detection of the patterns in these three cases was the breakthrough in our study, and we infer two valid species. We can reject the alternative hypothesis that the distinct mtDNA clades are solely caused by genetic contacts of some metapopulations with the closely related *cerdaniensis* song group for *C*. *brevipennis* and *C*. *petryi*.

In contrast to the *C*. *brevipennis*/*C*. *petryi* example, the data do not indicate that *C*. *b*. *brevipennis* and *C*. *b*. *hippolaidica* ssp. n. are evolving separately. We describe them as subspecies. They may be an example of geographically allopatric clades (see [10]) that were separately evolving but later came back into secondary contact before reproductive isolation was complete. At some point in time (or at multiple time points), these two lineages met in central Italy and likely mixed over large areas (Figs 3B and 4B). As with many subspecies, it is not easy to draw the spatial limits of the two taxa. It could be argued that *C*. *b*. *hippolaidica* ssp. n. is endemic to the Monti della Daunia (“MD”), but our distribution data suggests that such a limit would not reflect the most natural system, at least not in the present day. The region between the two national parks Monti Sibillini and Majella acts as a barrier where subspecific exchange is hindered by the presence of *C*. *sibillae* from the *cerdaniensis* group (i.e. competitive exclusion).

Additional contact zones may exist in the south-eastern Alps (*C*. *b*. *brevipennis* and *C*. *petryi*) and in southern France (*C*. *b*. *litoralis* and *C*. *petryi*) but not in the Northern Apennine where *C*. *b*. *brevipennis* and *C*. *petryi* are clearly separated. These potential hybrid zones are small compared to the wider distributional area (Fig 10) and, therefore, are not influential in our taxonomic decisions. Only two populations (Torreilles “TO”, Travesio “TR”) among 31 showed intermediate signals in acoustics at the species level (Figs 5 and 6).

*Cicadetta b*. *litoralis* ssp. n. is certainly more than an ecotype with exceptional habitat requirements; it appears to have evolved unique attributes that could lead it to speciate along a separate trajectory. Our data supports a close relationship to *C*. *b*. *brevipennis* and *C*. *b*. *hippolaidica* despite the spatial separation. However, some elements are similar to *C*. *petryi* (IED_S_L_ minimum, colouration of basal junction of anal veins). The genetic distance to *C*. *b*. *brevipennis* implies that it is a very young taxon (Table 1 and Fig 3A). The remarkable ecological and phenotypic adaptations were possibly favoured by drift in a small founder population (e.g. [88,89]).

The *brevipennis* basic song pattern is relatively simple and provides only a few variables in the time and carrier frequency domains. Therefore, we were looking for additional potential traits and experimented with the power succession until we found indicative characters (Fig 2), which are presumably being used for the first time in cicada taxonomy. Acoustic differences between the taxa are generally small, but the sum of specific traits is convincing (Table 4). The three best song characters in the data set have 0, 3 and 4 individuals in the overlap for *C*. *petryi* and *C*. *b*. *brevipennis* (Fig 5) and are probably reliable on similar levels when applied to randomly sampled individuals. Combining these characters results in a well-supported separation of the two taxa (Fig 6) and may contain relevant acoustic information when communicating with conspecific versus non-conspecific females. There are other examples within European cicadas, where inherited acoustic specific-mate recognition systems are fine-tuned [2,29,90]. With one colouration character (basal junction of the anal veins) we assign only 4.5% of the specimens to the wrong group of taxa (normally dark: *C*. *b*. *brevipennis* and *C*. *b*. *hippolaidica*, normally yellowish: *C*. *petryi* and *C*. *b*. *litoralis*).

### Evolutionary hypotheses for the origin of the *C*. *cerdaniensis* and *C*. *brevipennis* group taxa

The complicated but regular evolutionary pattern between the *cerdaniensis* and the *brevipennis* song groups is unusual and intriguing. We suggest a possible explanation. This scenario combines the specific traits with traditional biogeographic models. Hereby, acoustic patterns serve as predictors of the most parsimonious evolution as well as indicators of recent hybridization [2,32,69]. Three pieces of evidence form the basis of our scenario: 1) The molecular data are structured spatially; 2) the genetic distances [91,92] between taxa of the three main clades (≈ mean 2.5%) suggest a radiation in the Pleistocene with its drastic climatic changes and severe impacts on populations [93–95]; and 3) the inter-group taxa pairs are likely to have been in contact.

**Fig 11:**
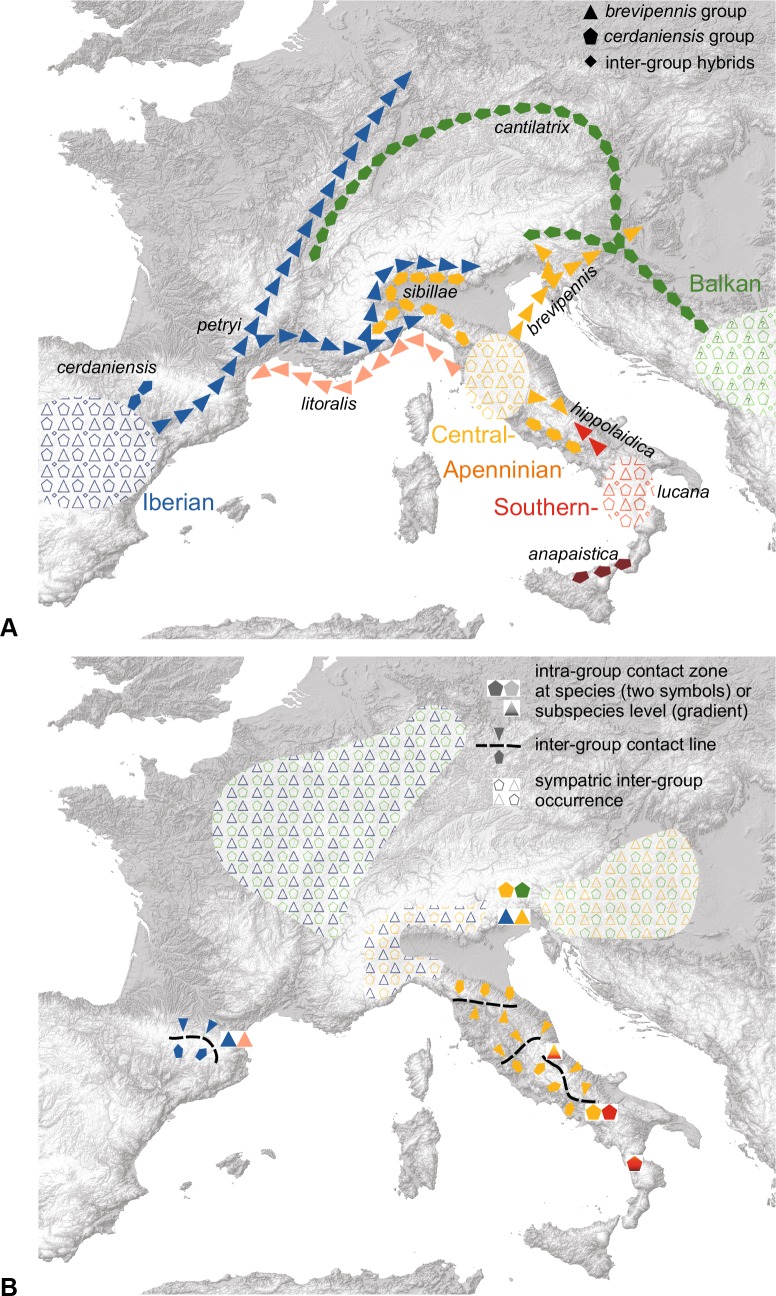
Biogeographical aspects within the *Cicadetta brevipennis* and *Cicadetta cerdaniensis* song groups. (A) Potential recolonization routes from supposed common inter-group Pleistocene refuges at the Iberian, Apenninian and Balkan Peninsulas. Glacial refuges are visualized schematically by coloured-outline hollow symbols. (B) Sympatric occurrences and contact zones of current populations. Blue = Iberian clade, red shades (pink–orange–red–brown red) = Apenninian clade, green = Balkan clade; triangles = *brevipennis* group, pentagons = *cerdaniensis* group.

Combining these three aspects, we hypothesize that widespread ancestors of the *cerdaniensis* and *brevipennis* groups were isolated from intra-group populations in different refugia during Pleistocene glaciation periods. European cicadas were likely displaced to the classic southern refugia [93–95] since they are thermophilic and most of them are connected to temperate forests or their margins. We suggest an Iberian refuge for *C*. *petryi* and *C*. *cerdaniensis* s. str., a central Apenninian for *Cicadetta b*. *brevipennis* and *C*. *sibillae*, a southern Apenninian for *C*. *b*. *hippolaidica* ssp. n., *C*. *a*. *anapaistica* and *C*. *a*. *lucana* and a Balkan for *C*. *cantilatrix* and possibly bulg (Figs 9 and 11A). During these long cold periods, genetic exchange with other intra-group metapopulations was at least hindered (within Apenninian Peninsula) or interrupted. Isolated metapopulations began to evolve specific traits. However, we speculate that they lived and reproduced in parapatry or even sympatry with populations belonging to the other song group. In these isolated populations, non-conspecific matings with sporadic gene flow could have taken place. This transfer led to mtDNA haplotypes (and presumably at least some nuclear genes) being shared among species and the mtDNA clades became diagnostic for refugia rather than species (see [19]). Even rare hybridization is enough to allow extensive introgression of mtDNA across species boundaries [8,20,96]. Mitochondrial capture in the absence of song introgression has been seen in other cicada taxa [32]. Incomplete lineage sorting as an alternative for non-monophyly predicts no consistent patterns with respect to geography [19,20,81] and is improbable for the main clades of our data set. We also suggest that the observed pattern is unlikely a result of a phenotypic convergence, even when taking into account, that astonishing cases have been documented for other insect groups but for morphological adaptations [97–99]. Phenotypic convergence is not able to explain why *brevipennis* and *cerdaniensis* song groups not only form taxa pairs but in many cases also share identical haplotypes. Additionally, the allopatric intra-group distributions are too perfect and the evolutionary costs of switching complex song patterns several times appear to be too high.

A similar explanation could be devised for the distribution of colouration characters, in particular the basal junction of the anal veins of the fore wing (Fig 9). Yellowish and dark phenotypes were displaced to the different refugia by glacial cycles. Probably, phenotypes–at least of one taxon–coming to the Apenninian Peninsula were often dark and rarely yellowish and the individuals from the Iberian Peninsula had a reciprocal frequency scale. We suggest that selection eliminated the rare phenotypes over thousands of generations and sporadic inter-group hybridization spread the dominant phenotypes to cohabiting taxa. In a southern Apenninian refuge, *C*. *a*. *lucana* or *C*. *b*. *hippolaidica* ssp. n. evolved lighter morphs, and obviously began to share them after episodic heterospecific reproduction.

Surprising is the fact, that the refugia scenario discussed above simultaneously took place in three to four different regions. The closely related ecological niches of *cerdaniensis* and *brevipennis* groups serve as a possible explanation, which inevitably leads to spatial contacts. Our data are paradoxical in the sense that on the one hand, gene trees and genetic distances were helpful to find cryptic species such as *C*. *sibillae* [2] and *C*. *petryi* and to roughly calibrate main clades. On the other hand, introgression affects our system so thoroughly that genetic distances are difficult to interpret in the whole *cerdaniensis*-*brevipennis* group. We cannot assess in which directions gene flow occurred or whether it occurs bidirectionally or unidirectionally. Thus, we also do not know which haplotypes are more original for the one or the other song group (see [20]). We do not even have evidence that *cerdaniensis* and *brevipennis* group ancestors are sister taxa.

All elements of our scenario are documented in other animal groups but as far as we know not in combination. Most similar to our scenario we found the gall wasp system (genus *Andricus*) described by Nicholls *et al*. [19] in which the mtDNA haplotypes of four hybridizing species cluster in multiple clades indicative of three common Pleistocene refugia. However, in this case, speciation during isolation in separate refugia did not involve reproductively important traits and the prediction of refugial membership was not as perfect as in the *cerdaniensis-brevipennis* groups, especially not for specimens collected far from the core refuge; e.g. our *C*. *petryi* from Central Germany still have haplotypes closely related to endemic *C*. *cerdaniensis* from the Pyrenees. Similarly, north Iberian hare species possess the mtDNA of *Lepus timidus* Linnaeus, 1758, a species that retreated from this region to the Alps and northern latitudes at the end of the last ice age [100]. Postglacial mtDNA replacement was seen in the New Zealand cicada *Kikihia muta* (Fabricius, 1775) after it crossed from North Island to South Island, invaded the territory of a non-sister species and hybridized with it [32]. In North American chipmunks, Sullivan *et al*. [8] found two pairs of non-sister species where the mtDNA of one member of each pair had completely replaced that of the other member prior to the Pleistocene with the taxa in question showing no current evidence of gene flow.

### Postglacial biogeography

From the current geographic distribution (Fig 10) and the scenario above we can hypothesize potential postglacial recolonization routes (Fig 11A; compare [93–95,101]). *Cicadetta petryi* and *C*. *cantilatrix* were obviously successful dispersers. Apenninian lineages of both groups have conquered similar areas, and the presence of the Alpine barrier prohibited them from colonising more northern areas [2,93–95,101]. We propose that *Cicadetta b*. *brevipennis* and *C*. *b*. *litoralis* ssp. n. profited by temporary migration routes when coastlines moved seaward due to the enormous amount of water stored in the continental ice sheets in the Late Pleistocene [102–104] as known for other organisms (e.g. [89,105–107]). Later these coastal plains were inundated again and populations were isolated and went locally extinct.

Postglacial expansions led to secondary contacts with intra-song group taxa (Fig 11B). When reproductive isolation was incomplete, lineages hybridized (e.g. [95]). Such contact zones can be found in central and southern Italy today. These Apenninian taxa share mtDNA haplotypes which are likely a result of intra-group introgression (compare [83]): *C*. *sibillae* completely asymmetrically captured the mtDNA of *C*. *a*. *lucana* [2], while *C*. *b*. *brevipennis* drove the distinct haplotype of *C*. *b*. *hippolaidica* ssp. n. almost to extinction (Fig 9, red symbols). Therefore, in both song groups the central Apenninian haplogroups became dominant. Another zone, where lineages having supposedly different origins meet, is found in the south-eastern Alps. Interestingly, the Trentino-Veneto region serves as a suture zone for both song groups (compare [101,108–110]). The *brevipennis* group is notable since Apenninian *C*. *b*. *brevipennis* approached *C*. *petryi* from the east.

Where *brevipennis* and *cerdaniensis* song groups met postglacially they exhibit an unusual and puzzling distribution: Inter-group taxa pairs for which we predict common glacial refugia and origins are nowadays not distributed sympatrically but parapatrically or allopatrically (Fig 10, Fig 11B). Only a few isolated individuals contradict this rule. Parapatric inter-group distribution areas end sharply within a few kilometres in the Apennine Mountains, and we can define contact lines. When populations of the two groups occur in sympatry or even in syntopy they stem from different mtDNA geographical clades. Three known regions show such patterns of associations (Fig 11B). We currently do not have an explanation for this phenomenon.

### Current distribution, ecology and threat

Fig 10A shows probable assignments of all records in our database to the new *brevipennis* group taxa (approximately 500 records, 50% from France, 33% from Italy, S2 Table). Interestingly, the southern extents of the *brevipennis* group distribution in the Balkan, Apenninian and Iberian Peninsulas are at similar latitudes. For the Apennine Mountains, the ridge decreases here to lower altitudes and classically divides the fauna into a central and a southern sector with changing species communities [2,108,110].

Like many other species of the *C*. *montana* complex, *brevipennis* song group taxa prefer ecotone habitats, e.g. between extensively cut or grazed open land and sparse woodlands. Often *brevipennis* group taxa sing in lower substrates (small bushes, herb layer) than is usually known for *C*. *montana* s. str. or for the *cerdaniensis* song group [39,40,74,77]. *Cicadetta brevipennis* group species tend to live in drier habitats than *cerdaniensis* group species. However, the ecological differences between the *cerdaniensis* and the *brevipennis* groups are generally small and, as mentioned above, syntopic populations do occur. Indeed, the habitat preferences among the *C*. *brevipennis* subspecies are remarkable and an additional diagnostic trait (Table 4).

Our discovery of four different taxa within the former *C*. *brevipennis* species provides evidence useful for the re-evaluation of endangerment potential [38–40,77]. All newly recognized taxa are restricted to rather small or dissected distributional ranges, even *C*. *petryi*. The highest priority taxon is *C*. *b*. *litoralis* ssp. n., a subspecies that seems critically endangered. One of the two known localities (Alénya) has already been destroyed due to land use intensification. We hope that this subspecies is present in coastal Spanish habitats and that the undetermined populations in the French Rhone delta also belong to *C*. *b*. *litoralis* ssp. n. (Fig 10, black dots). The habitat in Torreilles should be protected immediately. Second in priority would be the *C*. *b*. *hippolaidica* ssp. n. population from the Monti della Daunia. It exhibits the purest and most extreme characters of this lineage. At third priority are the completely fragmented local populations of *C*. *petryi* remote from the core populations in southern France. It is rare with regionally restricted habitat requirements in Switzerland, Germany and Italy as well as in northern France [40,65,74,77]. Many of these isolated populations appear as relicts of a postglacial warmer period with tens of kilometres between them. *Cicadetta b*. *brevipennis* needs to be investigated more deeply in the Balkans and in Eastern Europe to judge its distributional area and the potential threats. It seems not to be endangered in central Italy and Slovenia. Last but not least, the Black Sea lineage (bulg) should be investigated in more detail, checking to see if it forms another endemic and possibly endangered species or, for example, a peripheral *C*. *b*. *brevipennis* metapopulation with some individuals possessing the mitochondrial genome of *C*. *cantilatrix*.

## Conclusions

We resolve an intricate pattern of mtDNA haplotype distributions by searching for congruent traits from different data sources in order to draw taxonomic conclusions and present a plausible biogeographic history. The system is easiest to understand when we attach the highest importance to the reproductively most relevant traits such as distinct song patterns. The former polyphyly is divided into the *brevipennis* and *cerdaniensis* song groups, and subtaxa of both groups are now much easier to comprehend geographically and taxonomically. We chose a system-adapted hierarchic approach, continually and consequently searching for lines of evidence of separately evolving metapopulation lineages versus partially isolated geographic varieties. The taxonomic result was a paradox: introgression helped to find cryptic species. Our approach required the availability of good distributional data and illustrates the importance of more integrated species delimitation procedures.

## Supporting Information

S1 Fig Power differences between phrases 1 and 2 in a spatial context. (PDF)

S2 Fig Maximum likelihood mtDNA phylogeny (COI and COII) with bootstrap values from RAxML analysis for the *Cicadetta brevipennis* group. (PDF)

S3 Fig Maximum likelihood mtDNA phylogeny (COI and COII) with bootstrap values from RAxML analysis for the *Cicadetta brevipennis* and *Cicadetta cerdaniensis* groups. (PDF)

S1 Table Spatial origin of the specimens analysed acoustically, morphologically or molecularly. (PDF)

S2 Table Database of the records of the *Cicadetta brevipennis* group. (XLSX)

S3 Table List of collecting permits within the *Cicadetta brevipennis* group distribution. (PDF)

S1 Text Nomenclature of *Cicadetta brevipennis*. (PDF)
