# Supplementary material for: Complex within a Complex: Integrative Taxonomy Reveals Hidden Diversity in Cicadetta brevipennis (Hemiptera: Cicadidae) and Unexpected Relationships with a Song Divergent Relative
Source: PLoS One. 2016 Nov 16;11(11):e0165562. doi: 10.1371/journal.pone.0165562 (PMC5112989; doi:10.1371/journal.pone.0165562)
Supplement: S1 Fig — (PDF) [file pone.0165562.s001.pdf]

S1 Figure

Hertach et al., 2016: *Cicadetta brevipennis* Integrative Taxonomy

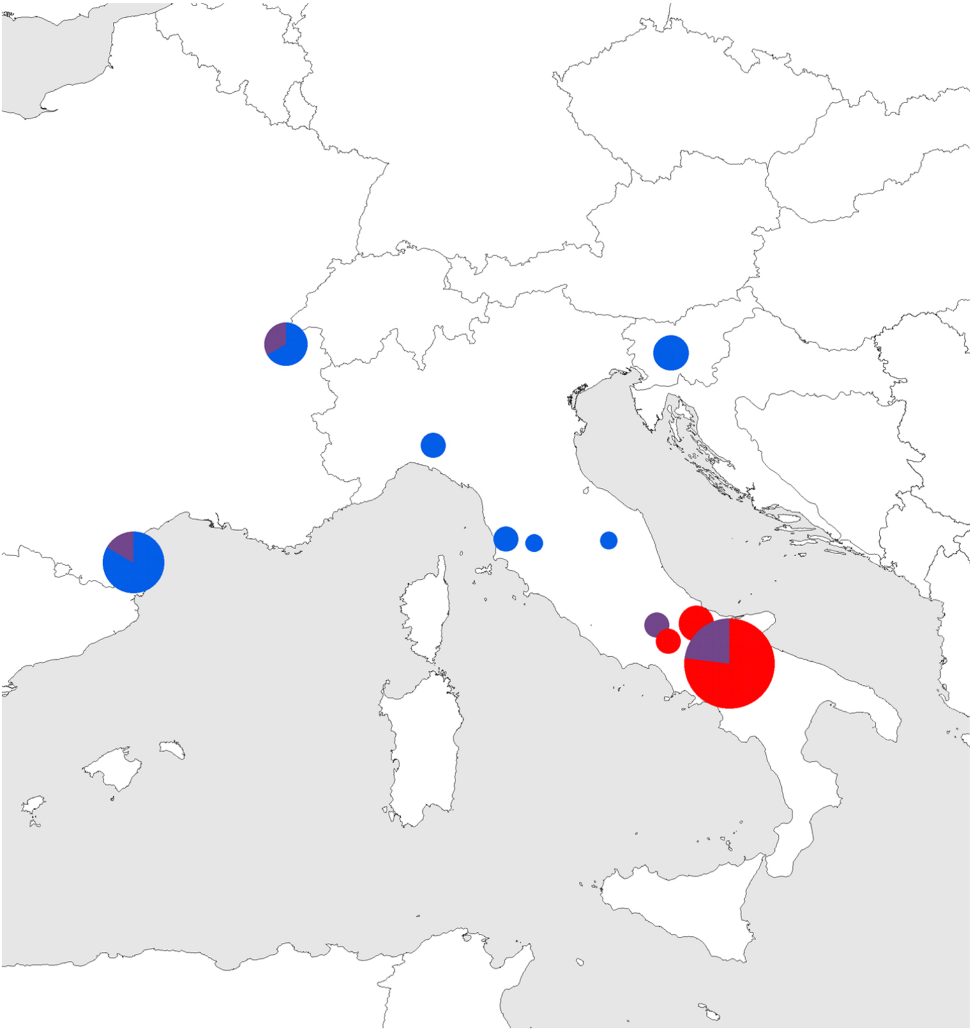

Power differences between phrases 1 and 2 ($EP_{L/5}$ - EP_{2L}) in a spatial context.

Significant differences between *Cicadetta brevipennis hippolaidica* ssp. n. and other taxa. Blue > 12.3 dB, red < 11.7 dB, violet = overlap. Size of circles relative to the number of investigated individuals (n_{max} = 13).
