# Supplementary material for: Complex within a Complex: Integrative Taxonomy Reveals Hidden Diversity in Cicadetta brevipennis (Hemiptera: Cicadidae) and Unexpected Relationships with a Song Divergent Relative
Source: PLoS One. 2016 Nov 16;11(11):e0165562. doi: 10.1371/journal.pone.0165562 (PMC5112989; doi:10.1371/journal.pone.0165562)
Supplement: S2 Fig — (PDF) [file pone.0165562.s002.pdf]

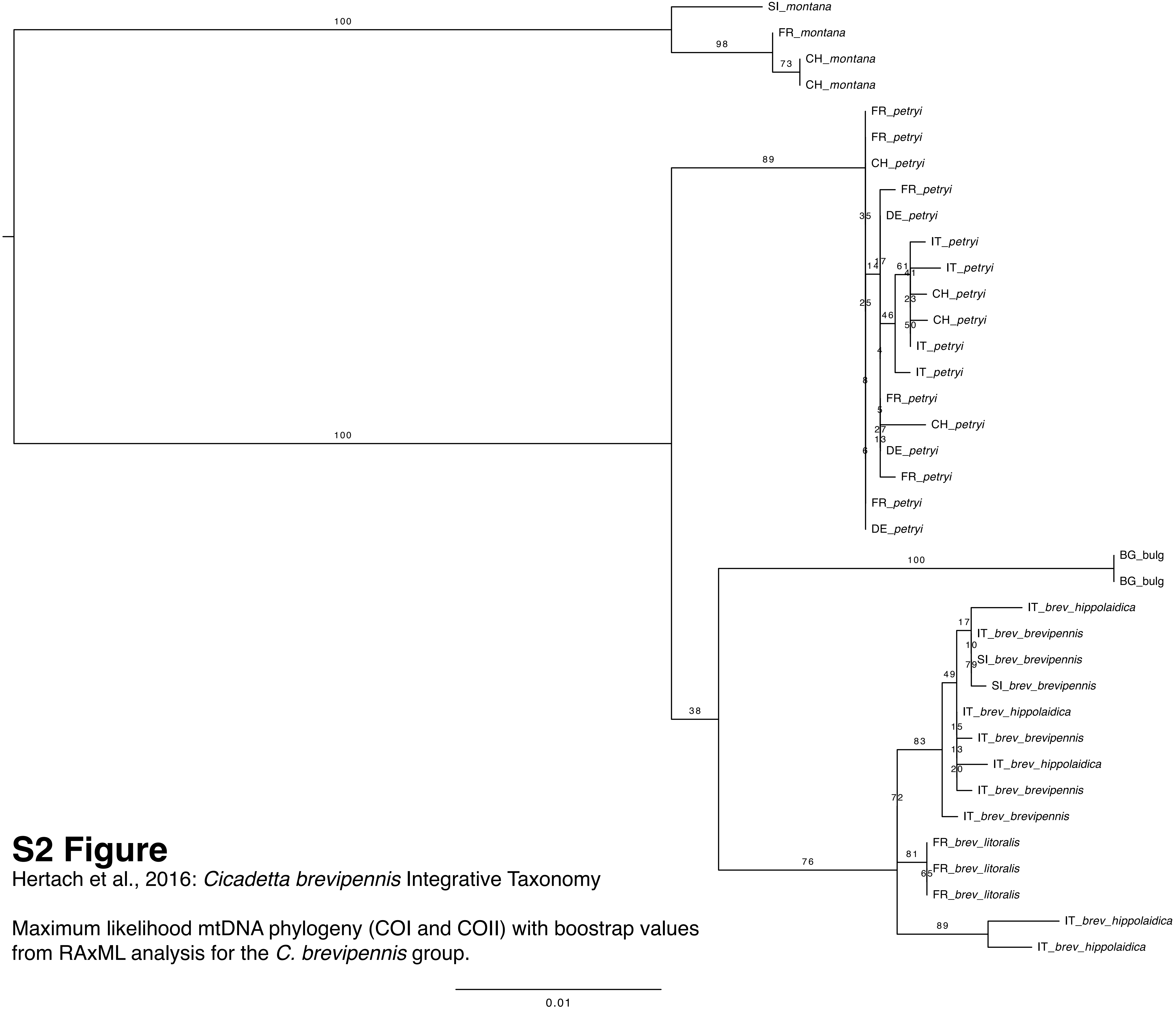


# S2 Figure

Hertach et al., 2016: *Cicadetta brevipennis* Integrative Taxonomy

Maximum likelihood mtDNA phylogeny (COI and COII) with boostrap values from RAxML analysis for the *C. brevipennis* group.
