# Supplementary material for: Complex within a Complex: Integrative Taxonomy Reveals Hidden Diversity in Cicadetta brevipennis (Hemiptera: Cicadidae) and Unexpected Relationships with a Song Divergent Relative
Source: PLoS One. 2016 Nov 16;11(11):e0165562. doi: 10.1371/journal.pone.0165562 (PMC5112989; doi:10.1371/journal.pone.0165562)
Supplement: S1 Table — (PDF) [file pone.0165562.s004.pdf]

S1 Table. Hertach et al., 2016: *Cicadetta brevipennis* Integrative Taxonomy

Spatial origin of the specimens analysed acoustically, morphologically or molecularly (page 1).

Taxon	Location, Region	Political Region	Country	Altitude [m asl]	Lat.	Long.	Observer	Date	Collection	Number of analysed specimens: morphology	mtDNA	1. song phrase	2. song phrase	occurrence of the second phrase
Cicadetta brevipennis brevipennis	Glanz an der Weinstrasse	Styria	Austria	400	46.6560	15.5220	Kunz/Gogala/Trilar	diverse	Kunz	2		3		6
Cicadetta brevipennis brevipennis	Velika, Papuk	Požeško slavonska	Croatia	380	45.4624	17.6400	Trilar/Gogala	30.05.2012						2
Cicadetta brevipennis brevipennis	NW Melezzole	Umbria	Italy	680	42.6762	12.3281	Thomas Hertach	13.07.2011						1
Cicadetta brevipennis brevipennis	Poggie le Perazzete, Monte Amiata	Tuscany	Italy	870	42.8235	11.6453	Thomas Hertach	15.07.2011				2		3
Cicadetta brevipennis brevipennis	Monte Ceci, Colline Metallifere	Tuscany	Italy	310	43.1110	10.6525	Thomas Hertach	13.07.2012				1		2
Cicadetta brevipennis brevipennis	Fontazzi, Murlo	Tuscany	Italy	200	43.1677	11.2955	Thomas Hertach	08.07.2012					0.5	3
Cicadetta brevipennis brevipennis	S Bivio Ercole, Fiuminata	Marches	Italy	550	43.1731	12.8867	Thomas Hertach	06.07.2012				2	0.5	3
Cicadetta brevipennis brevipennis	Crevole, Murlo	Tuscany	Italy	290	43.1839	11.3720	Thomas Hertach	08.07.2012				1		2
Cicadetta brevipennis brevipennis	Busco, Cortona	Tuscany	Italy	590	43.2466	12.0973	Thomas Hertach	07.07.2012						2
Cicadetta brevipennis brevipennis	Foresta di Monterufoli	Tuscany	Italy	500	43.2488	10.7485	Thomas Hertach	14.07.2012	Hertach	2	2	1	1	2
Cicadetta brevipennis brevipennis	N Castellina Marittima	Tuscany	Italy	400	43.4217	10.5833	Thomas Hertach	11.07.2008						2
Cicadetta brevipennis brevipennis	W San Gimignano	Tuscany	Italy	530	43.4561	10.9512	Thomas Hertach	14.07.2012						2
Cicadetta brevipennis brevipennis	Colline Livornesi, Livorno	Tuscany	Italy	350	43.5117	10.4267	Thomas Hertach	13.07.2008	Hertach	1	1			
Cicadetta brevipennis brevipennis	Monte Acuto, Cantiano	Marches	Italy	760	43.5126	12.6554	Thomas Hertach	05.07.2012				1		4
Cicadetta brevipennis brevipennis	Colombara - Serravalle di Carda	Marches	Italy	590	43.5587	12.4608	Thomas Hertach	05.07.2012				1		4
Cicadetta brevipennis brevipennis	Passo dell'Incisa, Alfero	Emilia-Romagna	Italy	820	43.8575	12.0451	Thomas Hertach	04.07.2012						1
Cicadetta brevipennis brevipennis	E Corniolo, Casentino-Falterona	Emilia-Romagna	Italy	470	43.9051	11.8154	Thomas Hertach	04.07.2012						2
Cicadetta brevipennis brevipennis	Fruška gora: Bešenovački	Vojvodina	Serbia	215	45.1145	19.7131	Gogala/Trilar	04.06.2015					(1)	
Cicadetta brevipennis brevipennis	Golo, Stražar	Osrednjeslovenska	Slovenia	760	45.9125	14.5151	Tomi Trilar	18.06.2013				1		1
Cicadetta brevipennis brevipennis	Log, Lukovica	Osrednjeslovenska	Slovenia	330	46.0090	14.3590	Matija Gogala	diverse	PMSL	10	2	2	2	3
Cicadetta brevipennis brevipennis	Dol pri Hrastniku	Zasavska	Slovenia	400	46.1450	15.1141	Gogala/Trilar/Kapla	06.06.1999	PMSL	3		1		2
Cicadetta brevipennis hippolaidica	N San Bartolomeo in Galdo	Campania	Italy	590	41.4533	15.0385	Thomas Hertach	20.07.2010					1	1
Cicadetta brevipennis hippolaidica	Monte Sambuco, Monti della Daunia	Apulia	Italy	790-920	41.5311	15.0825	Thomas Hertach	diverse	NHMB/NMBE/Hertach	6	3	6	12	27
Cicadetta brevipennis hippolaidica	N Cerasuolo, Le Mainarde	Lazio	Italy	780	41.5953	14.0225	Thomas Hertach	03.07.2011						3
Cicadetta brevipennis hippolaidica	Lago Selva, Cardito, Le Mainarde	Lazio	Italy	930	41.6071	13.9758	Thomas Hertach	03.07.2011	Hertach	1		2	1	3
Cicadetta brevipennis hippolaidica	E Picinisco, Le Mainarde	Lazio	Italy	1200	41.6527	13.9037	Thomas Hertach	03.07.2011	Hertach	1				
Cicadetta brevipennis hippolaidica	S Pietrabbondante	Molise	Italy	960	41.7158	14.3767	Thomas Hertach	02.07.2011	NMBE	1				
Cicadetta brevipennis hippolaidica	NW Civitacampomarano	Molise	Italy	850	41.7972	14.6506	Thomas Hertach	01.07.2011				2	0.5	4
Cicadetta brevipennis hippolaidica	N Pescasseroli	Abruzzo	Italy	1260	41.8564	13.7847	Thomas Hertach	04.07.2011	NMBE/Hertach	2		1	1	2
Cicadetta brevipennis hippolaidica	N Torrebruna	Abruzzo	Italy	750	41.8846	14.5297	Thomas Hertach	01.07.2011	Hertach	3	2	1	1.5	5
Cicadetta brevipennis litoralis	Alénya	Pyrénées Orientales (66)	France	4	42.65XX	2.99XX	Stéphane Puissant	31.05.1999	Puissant	2				
Cicadetta brevipennis litoralis	Torreilles	Pyrénées Orientales (66)	France	2	42.76XX	3.02XX	Stéphane Puissant	diverse	MNHN/PMSL/Puissant/Hertach	21	3	4	6	5
Cicadetta brevipennis s.l. ("bulg")	Batovo	Dobrich Province	Bulgaria	120	43.4161	27.9414	Ilia Gjonov	23.06.2014	Hertach	2	2			2
Cicadetta brevipennis s.l. ("bulg?")	SE Meden Buk	Haskovo Province	Bulgaria	110	41.3697	26.0522	Gogala/Gjonov	06.07.2012						2
Cicadetta brevipennis s.l. ("bulg?")	E Melnik	Blagoevgrad Province	Bulgaria	520	41.5247	23.4224	Gogala/Trilar/Gjonov	18.06.2009						2
Cicadetta brevipennis s.l. ("bulg?")	NE Kalovo, Strandzha	Burgas Province	Bulgaria	160	42.1307	27.4930	Gogala/Trilar/Gjonov	22.06.2009						2
Cicadetta brevipennis s.l. ("bulg?")	Mladezhko, Strandzha	Burgas Province	Bulgaria	210	42.1517	27.3618	Gogala/Trilar/Gjonov	24.06.2009						2
Cicadetta brevipennis s.l. ("bulg?")	Rezervatie Padurea Hagieni, Albeşti	Constanţa	Romania	20	43.8024	28.4501	Gogala/Trilar/Popa	26.06.2004	PMSL	2				

S1 Table. Hertach et al., 2016: *Cicadetta brevipennis* Integrative Taxonomy

Spatial origin of the specimens analysed acoustically, morphologically or molecularly (page 2).

										Number of analysed specimens				
**Taxon**	**Location, Region**	**Political Region**	**Country**	**Altitude [m asl]**	**Lat.**	**Long.**	**Observer**	**Date**	**Collection**	morphology	mtDNA	1. song phrase	2. song phrase	occurrence of the second phrase
Cicadetta brevipennis s.l. (Hybrid?)	Travesio	Friuli–Venezia Giulia	Italy	470	46.2058	12.8590	Thomas Hertach	12.06.2010				1		1
Cicadetta brevipennis s.l. (Hybrid?)	Avaglio near Villa Santina	Friuli–Venezia Giulia	Italy	680	46.4280	12.9155	Thomas Hertach	08.06.2010	Hertach	1	1			
Cicadetta brevipennis	Cona di Selvapiana, Monti Lepini	Lazio	Italy	570	41.5720	13.1351	Thomas Hertach	24.07.2010				2		3
Cicadetta brevipennis	Forca di Penne, Villa Santa Lucia degli Abruzzi	Abruzzo	Italy	920	42.2827	13.8221	Thomas Hertach	06.07.2011						2
Cicadetta brevipennis	SE Campotosto, Monti della Laga	Abruzzo	Italy	1370	42.5366	13.4013	Thomas Hertach	11.07.2011				1		3
Cicadetta petryi	Ejulve-Villarluengo	Aragon	Spain	1480	40.7532	-0.6037	Stéphane Puissant	7./9.06.2015	Puissant	(7)			(2)	
Cicadetta petryi	Badebany, Fuilla	Pyrénées Orientales (66)	France	640	42.5749	2.3610	Thomas Hertach	15.06.2011	Hertach	1	1	3		3
Cicadetta petryi	Duilhac-sous-Peyrepertuse (castle)	Aude (11)	France	670	42.8700	2.5570	Stéphane Puissant	20.07.2001	Puissant	1				
Cicadetta petryi	Saint-Félix-de-l'Héras	Hérault (34)	France	630	43.8311	3.3065	Matthieu Aubert	11.07.2013	Hertach	1	1			
Cicadetta petryi	Châlet Reynard, Mt. Ventoux	Vaucluse (84)	France	1400	44.1510	5.3190	Stéphane Puissant	29./30.06.1998	Puissant	3				
Cicadetta petryi	Sérignan-du-Comtat	Vaucluse (84)	France	150	44.2033	4.8353	Puissant/Boulard/Yense	diverse	MNHN/Puissant	11				
Cicadetta petryi	Lagarde-Paréol	Vaucluse (84)	France		44.22XX	4.85XX	Michel Boulard	10.07.1978	MNHN	1				
Cicadetta petryi	Mostuéjouls, Gorges du Tarn	Aveyron (12)	France	410	44.2215	3.2225	Cédric Mroczko	07.07.2013	Hertach	1	1			
Cicadetta petryi	Col d'Anelle, Mercantour	Alpes-Maritimes (06)	France	1730	44.2730	6.8910	Hannes Baur	24.06.2012	NMBE	1	1			
Cicadetta petryi	Demafé, Aspres-sur-Buëch	Haut-Alpes (05)	France	785	44.5421	5.7784	Kevin Gurcel	30.06.2001	Gurcel	1				
Cicadetta petryi	Capanne di Cosola, Monte Lesima	Emilia-Romagna	Italy	1420	44.6679	9.2052	Thomas Hertach	22.06.2012				1		1
Cicadetta petryi	Corbesassi, Brallo di Pregola	Lombardy	Italy	1000	44.7077	9.2653	Thomas Hertach	04.07.2013				1		3
Cicadetta petryi	Monte d'Alpe/Passo del Penice	Lombardy	Italy	1050	44.8022	9.3149	Thomas Hertach	03.07.2013	Hertach	4	3		1	3
Cicadetta petryi	Bouthoux, Clelles	Isère (38)	France	820	44.8131	5.6291	Kevin Gurcel	15.07.2011	Gurcel	1				
Cicadetta petryi	Les Calloudes, Montmélian	Savoie (73)	France	500	45.5087	6.0475	Kevin Gurcel	12.07.2013	Gurcel	1				
Cicadetta petryi	Combefolle, Saint-Jean-de-la-Porte	Savoie (73)	France	770	45.5601	6.1116	Kevin Gurcel	14.06.2009	Gurcel	1				
Cicadetta petryi	SE Epinel, Cogne	Aosta	Italy	1510	45.6214	7.3334	Thomas Hertach	14.06.2012	Hertach	2	1	1		
Cicadetta petryi	Pont El, Cogne	Aosta	Italy	900	45.6783	7.2207	Thomas Hertach	13.06.2012	Hertach	1		2		2
Cicadetta petryi	Le Rosay, Saint-Ferréol	Haut-Savoie (74)	France	530	45.7624	6.3206	Kevin Gurcel	09.06.2012	Gurcel	1				
Cicadetta petryi	Les Savioz, Marlens	Haut-Savoie (74)	France	830	45.7698	6.3321	Kevin Gurcel	diverse	Gurcel	2				
Cicadetta petryi	S Doues, Valpelline	Aosta	Italy	1120	45.8083	7.3067	Trilar/Hertach	10.06.2007						2
Cicadetta petryi	Monte Barro, Lecco	Lombardy	Italy	720	45.8288	9.3800	Thomas Hertach	02.07.2013						2
Cicadetta petryi	NE Valpelline	Aosta	Italy	1150	45.8300	7.3317	Trilar/Hertach	10.06.2007						1
Cicadetta petryi	Monte San Giorgio, Meride	Ticino	Switzerland	1040	45.9128	8.9491	Thomas Hertach	diverse	Hertach	8	2	3		7
Cicadetta petryi	Montagne des Princes, Val de Fier	Haut-Savoie (74)	France	900	45.9442	5.8782	Kevin Gurcel	diverse	Gurcel	3				
Cicadetta petryi	Le Rucher-Les Vorziers, Vanzy	Haut-Savoie (74)	France	320	46.0214	5.8714	Kevin Gurcel	12.06.2013	Gurcel	1				
Cicadetta petryi	Le Mont, Chaumont	Haut-Savoie (74)	France	600	46.0378	5.9570	Kevin Gurcel	18.06.2007	MNHN/PMSL/Puissant/Hertach	1				
Cicadetta petryi	Pougny, Pays de Gex	Ain (01)	France	360	46.1406	5.9343	Thomas Hertach	25.05.2011				1		2
Cicadetta petryi	Bois de Treulaz, Aire-la-Ville	Geneva	Switzerland	380	46.1880	6.0345	Thomas Hertach	20.05.2011	Hertach	1				3
Cicadetta petryi	Mauregard, Russin	Geneva	Switzerland	380	46.1969	6.0270	Hertach/Trilar/Gurcel	diverse	PMSL/Hertach/Gurcel	4	1	1	1	4
Cicadetta petryi	Bois de la Grille, Vernier	Geneva	Switzerland	410	46.2145	6.0974	Trilar/Hertach	diverse	PMSL/Hertach	5		1	2	2
Cicadetta petryi	Les Vaux, Arnex-sur-Orbe	Vaud	Switzerland	480	46.6843	6.5195	Thomas Hertach	diverse	Hertach	6	1	2		4
Cicadetta petryi	Grasberg, Bergheim	Haut-Rhin (68)	France	280	48.2125	7.3586	Thomas Hertach	23.05.2009	Hertach	1	1	1		4
Cicadetta petryi	La Roche-Guyon, Haut-Isle	Val d'Oise (95)	France	75	49.0850	1.6420	Jérôme Sueur	29.06.2006	Puissant	3	1			
Cicadetta petryi	Frankenhausen, Kyffhäuser	Thuringia	Germany	150-210	51.3638	11.0985	Hertach/Petry	diverse	ZMHB/Hertach	5	3	3		4
