# Supplementary material for: Complex within a Complex: Integrative Taxonomy Reveals Hidden Diversity in Cicadetta brevipennis (Hemiptera: Cicadidae) and Unexpected Relationships with a Song Divergent Relative
Source: PLoS One. 2016 Nov 16;11(11):e0165562. doi: 10.1371/journal.pone.0165562 (PMC5112989; doi:10.1371/journal.pone.0165562)
Supplement: S3 Table — (PDF) [file pone.0165562.s006.pdf]

S3 Table. List of collecting permits within the *Cicadetta brevipennis* group distribution - Hertach et al., 2016: *Cicadetta brevipennis* Integrative Taxonomy

Area	Country	Authority	Receiver	Year	Identification/decision No.
Pirin (National Park)	Bulgaria	*Interacademic exchange between SAZU and PAS* in cooperation with Dr. Alexi Popov and Dr. Nikolay Simov (National Museum of Natural History, Sofia) and Dr. Ilia Gjonov (Department of Zoology and Anthropology of Sofia University "St. Kliment Ohridski")	Matija Gogala, Tomi Trilar	2008-2010	
Rila (National Park)	Bulgaria	*Interacademic exchange between SAZU and PAS* in cooperation with Dr. Alexi Popov and Dr. Nikolay Simov (National Museum of Natural History, Sofia) and Dr. Ilia Gjonov (Department of Zoology and Anthropology of Sofia University "St. Kliment Ohridski")	Matija Gogala, Tomi Trilar	2008-2010	
Strandzha (Natural Park)	Bulgaria	*Interacademic exchange between SAZU and PAS* in cooperation with Dr. Alexi Popov and Dr. Nikolay Simov (National Museum of Natural History, Sofia) and Dr. Ilia Gjonov (Department of Zoology and Anthropology of Sofia University "St. Kliment Ohridski")	Matija Gogala, Tomi Trilar	2008-2010	
Mt. Papuk	Croatia	Ministarstvo zaštite prirode i okoliša Republike Hrvatske	Matjia Gogala, Tomi Trilar	2012	KLASA: UP/I-612-07/12-33/28, URBROJ: 517-07-1-1-1-12-2
Mt. Sljeme	Croatia	Ministarstvo zaštite prirode i okoliša Republike Hrvatske	Matjia Gogala, Tomi Trilar	2012	KLASA: UP/I-612-07/12-33/28, URBROJ: 517-07-1-1-1-12-2
Kyffhäuser	Germany	Amt für Umwelt, Natur und Wasserwirtschaft, Sondershausen; in cooperation with Dr. Herbert Nickel, Göttingen	Thomas Hertach	2012-2014	IV.2.3 - 364.53.1/03_01_2012-nickel-Zikaden
Appennino Tosco-Emiliano	Italy	Ente Parco Nazionale	Thomas Hertach	2012	Prot. n. 710
Archipelago Toscano	Italy	Ministerio dell'Ambiente e della Tutela del Territorio e del Mare; Parco Nazionale	Thomas Hertach	2012	Prot. n. 1795
Dolomiti Bellunesi	Italy	Ente Parco Nazionale	Thomas Hertach	2010	Prot. 20100001011
Foreste Casentinesi - Monte Falterone	Italy	Sede legale del Parco Nazionale	Thomas Hertach	2012	Prot. 1015/2012 pos 11.2.1
Frignano	Italy	Ente di Gestione per i Parchi e la Biodiversità, Emilia Centrale; Parco Nazionale	Thomas Hertach	2012	no number (dated: 4 May 2012)
Gargano	Italy	Servizio Conservazione e Tutela della Natura; Parco Nazionale	Thomas Hertach	2010	autorizzazione n°8 del 20/04/2010
Gran Sasso - Monti della Laga	Italy	Ente Parco Nazionale	Thomas Hertach	2011	Prot. 0004126/01
Majella	Italy	Coordinamento Territoriale per l'Ambiente del Parco Nazionale	Thomas Hertach	2010/2011	Prot. 2493 and 3126
Monte Subasio	Italy	Comunità Montana dei Monti Martani, Serano e Subasio; Parco Regionale	Thomas Hertach	2011	Prot. 0006687
Monti Sibillini	Italy	Ente Parco Nazionale	Thomas Hertach	2011	Prot. n. 2051
Monti Simbruini	Italy	Ente Regionale di Diritto Pubblico; Parco Naturale Regionale	Thomas Hertach	2011	Prot. 1121
Sirente Velino	Italy	Corpo Forestale dello Stato; Parco Regionale	Thomas Hertach	2011	Prot. n. 597
the Abruzzi	Italy	Ente Automono Parco Nazionale	Thomas Hertach	2010/2011	Prot. 0001703/2010 and 0001728/2011
Padurea Hagieni (nature reserve)	Romania	*Interacademic exchange between SAZU and ASR* in cooperation with Prof. Dr. Laszlo Rakosy (Cluj University) and accompanied by Dr. Valentin Popa (Zoological Museum of Cluj University)	Matija Gogala, Tomi Trilar	2004/2008	
Fruška Gora	Serbia	Natsionalni Park; supervised by Dragiša Savić, member of staff	Matija Gogala, Tomi Trilar	2015	
Triglav (National Park)	Slovenia	Slovenian Environment Agency (ARSO)	Matjia Gogala, Tomi Trilar	ongoing	ARSO No. 35409-19/00
Aragón	Spain	Instituto Aragones de gestion ambiental, Zaragoza	Stéphane Puissant	2014/2015	NIF 110366202768
