# Supplementary material for: Complex within a Complex: Integrative Taxonomy Reveals Hidden Diversity in Cicadetta brevipennis (Hemiptera: Cicadidae) and Unexpected Relationships with a Song Divergent Relative
Source: PLoS One. 2016 Nov 16;11(11):e0165562. doi: 10.1371/journal.pone.0165562 (PMC5112989; doi:10.1371/journal.pone.0165562)
Supplement: S1 Text — (PDF) [file pone.0165562.s007.pdf]

S1 Text
Hertach et al., 2016: *Cicadetta brevipennis* Integrative Taxonomy

Nomenclature of *Cicadetta brevipennis*

Fieber (1876) described *C. montana* var. *longipennis* and *C. montana* var. *brevipennis* in his comprehensive taxonomic work on European Auchenorhyncha, which was posthumously printed in a French translation by Reiber. The names '*longipennis*' and '*brevipennis*' are available (Art. 10.2, ICZN, 1999). Both taxa were classified as synonyms of *Cicadetta montana* (s. l.) for several decades. Gogala & Trilar (2004) regarded '*brevipennis*' as a valid taxon of the binary long-short-echeme song pattern, and raised the variety to species rank. They mainly referred to Slovenian populations (e.g., figure with oscillogram) where the OTU brev was found within our study. Since Fieber did not describe songs, we have learned after the publication by Gogala & Trilar (2004) that Fieber's detailed but complicated morphological descriptions of both, '*brevipennis*' and '*longipennis*', more or less apply to all seven acoustically defined species subsequently discovered in this complex. His intraspecific boundaries are within the interspecific variability. Thus, *C. montana* var. *brevipennis* sensu Fieber and *C. brevipennis* sensu Gogala & Trilar is perhaps not the same. It is even likely that in Fieber's original type series specimens of multiple song-defined taxa have been treated.

Fieber did not provide a precise type locality but described the distribution area as (together with *C. montana* var. *longipennis*): "Distributed in whole Europe, rarer in northern parts, additionally found on the Crimea peninsula, the Ural Mountains and Siberia". His unpublished colour plates preserved in the National Museum of Natural History Paris (Soulier-Perkins, pers. comm.) do not contribute important new morphological information; the basal junction of the anal veins is not visible. At least a notice indicates that one drawn specimen originates from the Graz region in Austria (Styria) where OTU brev in fact occurs (Trilar & Holzinger, 2004 and this work: population at Glanz an der Weinstrasse (GL); see Fig 5C, in main article). Two other species have been found (*C. montana* s. str., Trilar & Holzinger, 2004) or can be expected (*C. cantilatrix*) in Styria with respect to biogeographical evidence. However, Fieber's description does not match our OTU brev specimens (see below) nor our *C. cantilatrix* from the whole distribution area. In both taxa, more or less the same characters are either predominantly appropriate or rarely true. *Cicadetta montana* s. str. seems to be congruent with Fieber's *Cicadetta megerlei* and can therefore be excluded from the debatable species.

The name *Cicadetta brevipennis* sensu Gogala & Trilar has been accepted and consequently used by subsequent entomologists during the last ten years (e.g., Trilar & Holzinger, 2004; Puissant, 2006; Boitier & Brugel, 2006; Hertach, 2007; Sueur & Puissant, 2007; Hugel *et al.*, 2008; Meineke, 2012; Delorme *et al.*, 2015; Gurcel, 2015). A type specimen has never been reported in literature. According to Melichar (1896), Handlirsch (1901) and Horn *et al.* (1990) the Fieber collection was given to Reiber, Puton, Lethierry and Noualhier and then came to the National Museum of Natural History Paris, the Museum of Natural History Vienna and the Royal Belgian Institute of Natural Sciences Brussels. We checked these collections ourselves and with the help of the curators and found two debatable specimens in Paris and Vienna.

The Paris specimen found in the Puton collection is labelled ”montana/Smyrna” (= Izmir, Turkey). Asia Minor has never been mentioned by Fieber as part of the geographic range. We conclude that Fieber did not know this specimen and it was probably Puton himself who added a typed label listing all the synonyms of *Cicadetta montana*, and ending with “var. *brevipennis* Fieb.”.

The Vienna specimen (Fig 8B, in main article) is without location but labelled with “*Cicadetta montana* var. *brevipenis*” (with one ‘n’!) and “*brevipennis* det. Fieber” (together with a putative *Cicadetta montana* var. *longipennis* specimen from Mehadia in Romania). The former label could be written by Fieber, the latter by Lethierry (see copies of handwritings in Horn *et al.*, 1990).

We extracted around 15 morphological characters, which Fieber provided to distinguish between *C. montana* var. *longipennis* and var. *brevipennis* first from the Reiber translation and then controlled it with the original unpublished handwritten German text kept in the National Museum of Natural History Paris (Soulier-Perkins, pers. comm.). Since no constitutive characters exist or were provided by Fieber, we generally did not weight the characters. The concordance between the putative Vienna type and Fieber’s description was low. We considered 36 to 47% of the characters as discordant, 8 to 43% as ambiguous and 29 to 54% as fitting the description. The range results from weak characters, which were independently evaluated by the first four authors of the study. Obvious characters do not fit such as a) the lateral part of the pronotal collar straight instead of “rounded”, b) in the fore wing, the outer rim of the costal vein with different colouration than the radial and subcostal veins instead of “monochrome”, c) the apical part of the operculum with a larger instead of “marginal portion yellowish” and d) the median lobe of uncus rather long-oval instead of “short and almost semi-circular”. These four characters are also easily visible on the unpublished colour drawings (National Museum of Natural History Paris; Soulier-Perkins, pers. comm.) and the Vienna specimen fits much better with the ‘*longipennis*’ plate and description. Though, it is also unlikely that the Vienna ‘*longipennis*’ and ‘*brevipennis*’ specimens have been interchanged. The concordance between the ‘*longipennis*’ specimen and the description would then decrease and in the ‘*brevipennis*’ specimen other characters would no longer fit. Fieber also stressed (1876: p. 88, description of *C. montana*) that among the considered material of both varieties some have “aberrant *one-sided* fused” median and cubitus anterior veins, but the Vienna specimen has an exceptional long fusion even on both sides (Fig 8B/F, in main article). A specimen so poorly fitting the description can scarcely be regarded as a type.

Another uncertainty arises from the spelling of the names ‘*brevipennis*’ (short-winged) and ‘*longipennis*’ (long-winged). Fieber’s description does not make any statement about the wing shape. It was only Schumacher (1924) who suggested that the wing ratio was a good character to use to distinguish between his ‘*brevipennis*’ and ‘*longipennis*’ groups. Fieber’s (1872) published list and handwritten original works (labels, unpublished texts and illustrations) suggest that his intention was to distinguish between *C. montana* var. *brevipenis nomen nudum* (short genitalia, probably: short median lobe of uncus and/or pseudoparameres) and *C. montana* var. *longipenis nomen nudum* (long genitalia), but Reiber translated the names incorrectly. By the way, the pseudoparameres can protrude more or less depending on the position and preparation of the specimen and this character is rather delicate in the manner used by Fieber. The spelling with two ‘n’ has been consequently used after 1876 and subsequent authors focussed more on wing shapes than on Fieber’s original characters; a vernacular French name has even established as “la Cigalette à ailes courtes” (≈ short-winged lesser cicada; Puissant, 2006; Deroussen *et al.*, 2014). ‘*Brevipenis*’ and ‘*longipenis*’ are both *nomina nuda* since Art. 12.1. (ICZN, 1999) is not satisfied in Fieber (1872) lacking a description, definition or indication.

Summarizing, in our opinion *Cicadetta montana* var. *brevipennis* sensu Fieber cannot be linked to a song defined species. For optimal stability and universality of the nomenclature, we favour petitioning the ICZN to set aside the doubtful Vienna specimen as a potential type and to designate a neotype of the OTU brev. For the neotype, we would like to choose a specimen from the population at Glanz an der Weinstrasse (GL) which is only 40 km away from Graz (ICZN, 1999; Art. 75.3.6). This population belongs to the same metapopulation as Slovenian brev and we can bring together *Cicadetta montana* var. *brevipennis* (sensu Fieber) and *Cicadetta brevipennis* (sensu Gogala & Trilar) in the most suitable way.

References

Boitier E, Brugel E. Les cigales en Auvergne: un essai de synthèse des connaissances (Hemiptera: Cicadidae). Arvernsis. 2006; 37-38: 7−12.

Delorme Q, Cury D, Bernier C. *Tettigetalna argentata* (Olivier, 1790) et les Cigales du groupe *Cicadetta* cf. *montana* (Scopoli, 1772) dans la moitié nord de la France: nouveaux éléments de répartition et d'écologie impliquant la conservation des espèces (Hemiptera Cicadidae). L'Entomologiste. 2015; 71(1): 31−40.

Deroussen F, Sueur J, Puissant S. Cigales de France. Cicadas of France. Paris: Muséum national d'Histoire naturelle. La sonothèque du Muséum. CD album; 2014.

Fieber FX. Katalog der europäischen Cicadinen nach Originalen mit Benützung der neuesten Literatur. Vienna: Carl Gerold's Sohn; 1872.

Fieber FX. Les Cicadines d'Europe d'après les originaux et les publications les plus récentes. Deuxième partie: Descriptions des espèces. Traduit de l'allemand par Ferd. Reiber. Revue et Magazin de Zoologie. 1876; 4: 11−268.

Gogala M, Trilar T. Bioacoustic investigations and taxonomic considerations on the *Cicadetta montana* species complex (Homoptera: Cicadoidea: Tibicinidae). An Acad Bras Cienc. 2004; 76(2): 316−324.

Gurcel K. Contribution à la connaissance des Cigales de France: première synthèse des observations et répartition des espèces pour le département de la Haute-Savoie (Hemiptera Cicadidae). L'Entomologiste. 2015; 71(4): 245−260.

Handlirsch A. Geschichte der Zoologie in Oesterreich von 1850-1900. III. Arthropoden, D. Insecten, Rhychoten. In: Hölder A, ed. Botanik und Zoologie in Österreich in den Jahren 1850 bis 1900. Vienna: Zoologisch-Botanische Gesellschaft, K. U. K. Hof- und Universitäts-Buchhändler; 1901. pp. 302−313.

Hertach T. Three species instead of only one: Distribution and ecology of the *Cicadetta montana* species complex (Hemiptera: Cicadoidea) in Switzerland. Mitteilungen der Schweizerischen Entomologischen Gesellschaft. 2007; 80: 37−61.

Horn W, Kahle I, Friese G, Gaedike R. Collectiones entomologicae. Ein Kompendium über den Verbleib entomologischer Sammlungen der Welt bis 1960. Berlin: Akademie der Landwirtschaftswissenschaften der Deutschen Demokratischen Republik; 1990.

Hugel S, Matt F, Callot H, Feldtrauer JJ, Brua C. Présence de *Cicadetta brevipennis* Fieber, 1876 en Alsace (Hemiptera, Cicadidae). Bull Soc Entomol Mulhouse. 2008; 64(1): 5−10.

International Commission on Zoological Nomenclature (ICZN). International Code of Zoological Nomenclature. 4th ed; 1999. Available: http://iczn.org. Accessed 16 June 2015.

Meineke T. Bergsingzikaden *Cicadetta cantilatrix* Sueur & Puissant, 2007, *Cicadetta brevipennis* Fieber, 1876 und *Cicadetta montana* s. str. (Scopoli, 1772) im mittleren Deutschland (Auchenorrhyncha, Cicadidae, Cicadettinae). Entomol Nachr Ber. 2012; 56(2): 133−142.

Melichar L. Cicadinen (Hemiptera-Homoptera) von Mittel-Europa. Berlin: Felix L. Dames; 1896.

Puissant S. Contribution à la connaissance des cigales de France: Géonémie et écologie des populations (Hemiptera, Cicadidae). Bédeilhac et Aynat: Association pour la Caractérisation et l’Etude des Entomocénoses; 2006.

Schumacher F. Auftreten der Bergzikaden in der Mark Brandenburg und Übersicht über die deutschen Formen (Hem. Homopt.). Berliner Entomologische Zeitschrift. 1924; 4: 329−334.

Sueur J, Puissant S. Similar look but different song: a new *Cicadetta* species in the *montana* complex (Insecta, Hemiptera, Cicadidae). Zootaxa. 2007; 1442: 55−68.

Trilar T, Holzinger WE. Bioakustische Nachweise von drei Arten des *Cicadetta montana*-Komplexes aus Österreich (Insecta: Hemiptera: Cicadoidea). Linz Biol Beitr. 2004; 36(2): 1383−1386.
